# Supplementary material for: Rapid expansion and visual specialisation of learning and memory centres in the brains of Heliconiini butterflies
Source: Nat Commun. 2023 Jul 7;14:4024. doi: 10.1038/s41467-023-39618-8 (PMC10328955; doi:10.1038/s41467-023-39618-8)
Supplement: Supplementary file 1 — Supplementary Information [file 41467_2023_39618_MOESM1_ESM.pdf]

Supplementary Information for

Rapid expansion and visual specialisation of learning and memory centres in the brains of Heliconiini butterflies

Antoine Couto*, Fletcher J Young*, Daniele Atzeni, Simon Marty, Lina Melo-Flórez, Laura Hebberecht, Monica Monllor, Chris Neal, Francesco Cicconardi, W Owen McMillan, Stephen H. Montgomery

* contributed equally

Correspondence to: s.montgomery@bristol.ac.uk

This PDF file includes:

1. Supplementary methods

1.1 Supplementary Method 1: Phylogenetic sampling across Heliconiini

1.2 Supplementary Method 2: Fixation

1.3 Supplementary Method 3: Neuropil staining

1.4 Supplementary Method 4: Neuronal tracing

1.5 Supplementary Method 5: Sectioning and mounting

1.6 Supplementary Method 6: Kenyon cell staining

1.7 Supplementary Method 7: Synapsin/Microglomeruli staining

1.8 Supplementary Method 8: Confocal image acquisition

1.9 Supplementary Method 9: Confocal image analysis

1.10 Supplementary Method 10: Electron microscopy sample preparation and imaging

1.11 Supplementary Method 11: Ecological data

1.12 Supplementary Method 12: Phylogenetic trees

2. Statistical analysis

2.1 Volumetric comparisons across Heliconiini

i) GLMs and allometric scaling

ii) phylogenetic modelling using BAYOU

iii) analysis of evolutionary rates and ancestral state reconstruction

2.2 Variation in Kenyon cell number

2.3 Variation in sensory input into the calyx

2.4 Testing ecological hypotheses

3. Behavioural experiments

3.1 Animals

3.2 Positive patterning

3.3. Biconditional discrimination

3.4 Long term memory

3.5 Statistical analysis

4. Supplementary Notes

4.1 Supplementary Note 1: Clade and pairwise differences in mushroom body scaling

4.2 Supplementary Note 2: Sex effects on mushroom body size

4.3 Supplementary Note 3: Additional results on mushroom body evolutionary rates

4.4 Supplementary Note 4: Additional neuroanatomical detail

4.5 Supplementary Note 5: Evolution of ventral lobula size

4.6 Supplementary Note 6: Variation in sensory innervation to the mushroom body calyx

4.7 Supplementary Note 7: Reconstructing pollen feeding evolution

4.8 Supplementary Note 8: Visual positive patterning learning in *Heliconius melpomene*

Other Supplementary Materials for this manuscript include the following, in a separate .xls file:

- Supplementary Data 1: Volumetric neuropil data for wild caught species: individual data
- Supplementary Data 2: Volumetric neuropil data for wild caught species: species means
- Supplementary Data 3: Kenyon cell number estimates
- Supplementary Data 4: Synapse number estimates
- Supplementary Data 5: Visual and olfactory calyx volumes
- Supplementary Data 6: Ecological data
- Supplementary Data 7: Behavioural data: positive patterning trials
- Supplementary Data 8: Behavioural data: biconditional discrimination trials
- Supplementary Data 9: Behavioural data: long term memory trials

1. Supplementary methods

1.1 Supplementary Method 1: Phylogenetic sampling across Heliconiini

Heliconiini are distributed across Central and South America and occur in a range of habitats from dry to wet forest, and from sea level to ~2500m. To ensure a thorough and even sampling across the phylogeny and avoid biased phylogenetic analyses, a series of sampling trips were performed to collect wild butterflies across the tribe. We are indebted to the environmental agencies in Costa Rica, Panama, French Guiana, Ecuador and Peru for permissions to carry out this work. Sampling of wild individuals (Supplementary Data 1) was focused on the following localities:

i) **Costa Rica:** Sampling was performed in 2015 at La Selva Biological Station (elevation 30-130m), Las Cruces Biological Station (elevation <20m), Le Leona eco-lodge on the edge of Corcovado National Park (elevation ~1000m), and Orosí (elevation ~1300m). Samples were collected under permit SINAC-SE-GASP-PI-R-2015. We thank the Organization for Tropical Studies at Las Cruces and La Selva, and Le Leona Eco Lodge for assistance in Costa Rica for support and assistance.

ii) **Panama:** Sampling was performed in 2012 and 2013 along Pipeline Road, Gamboa (elevation 60 m), which transects open to closed forest, and the nearby Soberanía National Park. Samples were collected under permits SEX/A-3-12, SE/A-7-13 and SE/AP-14-18. We are grateful to the *Heliconius* community at the Smithsonian Tropical Research Institute for valuable support during this period.

iii) **French Guiana:** Sampling was performed in 2016 at several urban sites round Cayenne and along roadsides (N1, N2, D5, D6) in forest edge habitats in the Arrondissement of Cayenne (elevation 0-150m). At the time of sampling no permits were required to sample outside National Parks in French Guiana.

iv) **Ecuador:** Sampling was performed at the Estación Científica Yasuní, in the Parque Nacional Yasuní, Orellana Province, Ecuador in November/December 2011 and September/October 2012 under permit collection no. 0033-FAU-MAE-DPO-PNY and exported under permit nos. 001-FAU-MAE-DPO-PNY and 006-EXP-CIEN-FAU-DPO-PNY. These were obtained from Parque Nacional Yasuní, Ministerio Del Ambiente, La Dirección Provincial de Orellana with the help of Estación Científica Yasuní and Pontificia Universidad Católica del Ecuador (PUCE). We are thankful to Á. Barragán, E. Moreno, P. Jarrín, and D. Lasso from the Estación Científica Yasuní, Pontificia Universidad Católica del Ecuador, and M. Arévalo from the Parque Nacional Yasuní Ministerio del Ambiente for assisting with collection and export permits. We are also grateful to F. Ramirez Castro for his assistance in the field in 2011. Additional collections were made from the forests around Vilcabamba (~1200m) and Balsas Canton (~500m) in Southern Ecuador in 2012 under permits provided by Ministerio del Ambiente del Ecuador to Dr Richard Merrill.

v) **Peru:** Sampling was performed in 2014 around the Escalera region near Tarapoto, Departamento de San Martín (elevation 300-1295 m), around Schucshuyacu (0-50m) on the Huallaga River in lowland forest to the east of Tarapoto, and along several higer eleveation sites to the northwest of Tarapoto: Abra Patricia at the San Martin/Amazonia boder (2350m), Catarata Yubilla, Cuispes (2100m), Catarata Gocta, Cocachimba (2300m), and roadside sites near Pedro Ruiz Gallo (1300m). Additional sampling was performed in Excalera in 2015. Samples were collected under permits 0289-2014-MINAGRI-DGFFS/DGEFFS, 020-014/GRSM/PEHCBM/DMA/ACR-CE, 040–2015/GRSM/PEHCBM/DMA/ACR-CE, granted to Dr Neil Rosser. We thank Neil Rosser, Ronald Mori Pezo and the Dasmahapatra group for assistance in Peru.

All individuals were collected using hand nets and kept alive in glassine envelopes until brain tissue could be fixed within a few hours of collection. Dissections (see below) were carried out at accommodation sites in the field. Body mass, length and wingspan were recorded as measures of body size and wings were kept in glassine envelopes as voucher specimens.

In addition to the wild caught samples, we obtained additional samples for selected species representing key lineages for subsequent analysis of the cellular composition and sensory domains of the calyx using pupae obtained from commercial supplies (Stratford Butterfly Farm, UK/London Pupae Supplies, UK/ Costa Rica Entomological Supplies, Costa Rica). Once dry, freshly eclosed butterflies were individually labelled and aged in a 2m^3 cage (2m x1m x 1m) in a controlled temperature room at 28°C with 80% humidity and a 12-hour day/night cycle. The cage was enriched with two plants, *Passiflora biflora* and *Lantana sp*., a natural food resource, with supplemental feeding using artificial feeders containing 30% sugar solution with ~10% bee collected pollen. For the neural tracing experiment and for synapse/microglomeruli quantification, the butterflies were sampled six to ten days after emergence, at which point they are considered behaviourally and sexually mature (Mallet 1981). For the quantification of Kenyon cells adult butterflies were sampled at least 24 hours after eclosion. as pilot data suggested major differences in mushroom body size are apparent at eclosion, suggesting the major shift in Kenyon cell production is completed by the adult stage.

1.2 Supplementary Method 2: Fixation

For all samples, following any additional procedures (see below), brains were dissected out in isotonic buffer-saline (HBS: 150 mM NaCl, 5 mM KCl, 5 mM CaCl2, 25 mM sucrose, 10 mM HEPES, pH 7.4) and fixed for 16 to 20 hours in zinc-formalin solution (ZnFA; 18.4 mM ZnCl2, 135 mM NaCl, 35 mM sucrose, 1% formalin), under gentle agitation at room temperature. The brain was then washed (3x 10 mins in HBS) and subsequently dehydrated into a solution containing 80% methanol and 20% dimethylsulfoxide (DMSO) for 2 hours, before storage in 100% methanol at -20°C. When ready to process, samples were brought back at room temperature and rehydrated in bath series of decreasing methanol concentration (90%, 70%, 50%, 30%, 0% in 0.1 M Tris buffer, pH 7.4, for 10 mins each).

1.3 Supplementary Method 3: Neuropil staining

For neuropil staining we followed Ott's (1) protocol. Mono-clonal anti-synapsin antibody (mouse anti-SYNORF1: 3C11, DSHB, RRID:AB_2315424), which bind a vesicle-associated protein at presynaptic site, was used to reveal the neuropil structure of the brain in all samples. To prevent antibodies from unspecific binding, brains were first incubated for 2 hours in a blocking solution (NGS-PBSd) containing 5% of normal goat serum (NGS: G9023, Sigma-Aldrich) in 0.1 M phosphate-buffered saline with 1% DMSO (PBSd). The primary antibody 3C11 (anti-SYNORF1) was then applied at a 1:30 dilution, in PBSd-NGS for 3.5 days at 4°C under agitation. Non-bound antibodies were rinsed out using three consecutive baths of PBSd solution (2h each) before incubation with Cy2-conjugated secondary antibody (Cy2 goat anti-mouse IgG: 115-225-146, Jackson ImmunoResearch, RRID: AB_2307343) diluted at 1:100 in PBSd-NGS for 2.5 days at 4°C. The brains were then dehydrated in bath series of increasing glycerol concentration (1%, 2%, 4% for 2 hours each, and 8%, 15%, 30%, 50%, 60%, 70%, 80% for 1 hour each) in 0.1 M Tris buffer with 1% DMSO, followed by 3 washes in 100% ethanol (30 mins each). The brain tissues were clarified in methyl salicylate (M6752, Sigma-Aldrich) for 30 mins before being transferred in fresh methyl salicylate, which was used as storage and mounting medium.

1.4 Supplementary Method 4: Neuronal tracing

To measure the volume of visual and olfactory areas within the mushroom bodies, projection neurons from primary sensory neuropils (the antennal lobe and the optic lobe) were differentially labelled using fluorescent tracers. Butterflies were immobilized in custom-made holders with the head isolated from the rest of the body using a plastic collar placed at the neck. To prevent the solution from leaking, a waterproof area was built by applying beeswax, melted at low temperature, around the head. The head capsule was then opened and the brain was exposed under a ringer solution (150 mM NaCL, 3mM $CaCl_2$, 3 mM KCL, 2 mM $MgCl_2$, 10 mM Hepes, 5 mM glucose, 20 mM sucrose) so that the tracheal sac and glands above the sensory neuropils could be removed. Under filtered illumination, the tip of pulled glass capillaries (G100-4, Warner Instruments) was loaded with crystals of dextran-conjugated dyes; either dextran-tetramethylrhodamine (fluoro-ruby: 10,000 MW, D1817, Thermo Fisher Scientific) or dextran-Alexa fluor 647 (10,000 MW, D22914, Thermo Fisher Scientific) mixed in 2% Bovine serum albumin (BSA). The loaded tip was either injected into the target neuropil(s), initially the mushroom body calyx, then subsequently the antennal lobe or in the caudal junction between optic lobe and the central brain (aiming for output neurons of the ventral lobe of the lobula), which were identified as major input sites to the calyx (Figure S8, see below). The glass capillary was kept in this position until the dye crystal dissolved so that neurons were imbued with the fluorescent tracer. After each injection, extra dye was removed by repeatedly washing the brain with Ringer solution, and absorbing the liquid with thin paper wipes. The heads were then covered with fresh Ringer solution and the butterflies were kept in the holders overnight in the dark. The next day, the brains were dissected out and preserved (as described in 1.2), for later background staining of the neuropils (as described in 1.3).

1.5 Supplementary Method 5: Sectioning and mounting

To visualize the cell bodies of Kenyon cells and calycal synapse density at high magnification, the butterfly brains were physically sectioned to overcome limitations of the working distance of high magnification lenses. Preserved samples were rehydrated (as described in 1.2) and embedded in 5% low melting point agarose (16520-050, Thermo Fisher Scientific). The brains were sliced horizontally along the anterior-posterior axis, in frontal sections with a thickness of 80 µm using a vibratome (VT 1000 S, Leica Biosystems, Wetzlar, Germany). The sections were collected and stained following 2 different procedures (described in 1.6 for Kenyon cells or in 1.7 for synapses/microglomeruli). After the staining procedure, brain sections were kept in 60% glycerol overnight, then mounted on microscope slides in 80% glycerol and surmounted by cover slips which were sealed with commercial nail polish.

1.6 Supplementary Method 6: Kenyon cell staining

To identify Kenyon cells over non-neuronal cells, cell nuclei were stained in combination with a specific marker of neuronal membrane, the anti-peroxidase antibody (HRP: Rabbit anti-horseradish peroxidae, P-7899, Sigma-Aldrich, RRID:AB_261181). Brain slices were first permeabilized in PBS containing 2% of Triton X-100, for 30 mins at room temperature. Slices were then blocked in PBS-NGS (PBS with 5% normal goat serum) with 0.2% Triton (PBSt-NGS thereafter). The brain tissues were probed with rabbit anti-HRP applied at a dilution of 1:10 000, in PBSt-NGS for 3 days at 4°C, under a constant agitation. Next, the slices were washed in PBSt (3 x 30 minutes), to remove residual unbound antibodies, and subsequently stained with Cy3-conjugated secondary antibody (Cy3 goat anti-rabbit IgG: 111-165-144, Jackson ImmunoResearch, RRID: AB_2338006), diluted at 1:200 in PBSt-NGS. After 3 days of incubation at 4°C, the brain sections were washed in PBSt (3x 30 mins) and stained with 1:1000 DAPI (D9542, Sigma-Aldrich) for 30 mins at room temperature. Extra reagent was then washed out and the brain slices were mounted on microscope slides (as described in 1.5).

1.7 Supplementary Method 7: Synapse/Microglomeruli staining

To assess the presynaptic boutons density within the calyx, brain slices were stained using anti-SYNORF1 antibody, or a combination of anti-SYNORF1 and Alexa-conjugated phalloidin which selectively label F-actin filament allowing to reveal dendritic profiles of Kenyon cells. For six species (Figure 3D) we focused solely on synaptic density using automated synapse counting of ZnFA fixed, anti-SYNORF1 stained sections (see 1.9), but we additionally stained using both primary antibodies for two species (Figure 3E; *H. charithonia* and *D. iulia*) to manually count microglomeruli, synaptic complexes containing presynaptic boutons and surrounding post synaptic Kenyon cell dendrites (2). As methanol destroys the quaternary structure of actin proteins, phalloidin stained samples were first fixed in a solution of methanol-free formaldehyde (18.4 mM ZnCl2, 135 mM NaCl, 35 mM sucrose, 1% formaldehyde) to preserve its native conformation. After fixation, both sets of samples were sliced (as described in 1.5) and stained as follows. Brain slices were permeabilized in PBS containing 2% Triton for 10 mins, and subsequently washed in PBSt (2x 10 mins). The samples were then blocked for 2 hours in PBSt-NGS and probed with anti-SYNORF1 antibody (3C11) at 1:30 in PBSt-NGS for 4

days at 4°C. After 3 consecutive washes in PBSt (3x 2h), the slices were stained in Alexa 568-conjugated secondary antibody (Alexa 568 goat anti-mouse IgG: A-11004, Thermo Fisher Scientific, AB_2534072) diluted at 1:200, and Alexa 488 phalloidin (A12379, Thermo Fisher Scientific) diluted at 0.5 units/mL in PBSt-NGS, for 7 days at 4°C. Finally, the slices were washed in PBSt (3x 20 mins) and mounted on microscope slides (as described in 1.5).

1.8 Supplementary Method 8: Confocal image acquisition

For whole mount brains used to quantify neuropil volumes, clarified samples (see 1.3) were mounted in wells drilled into custom made 3mm thick aluminium-slides, filled with methyl salicylate, and sealed with cover slips on both sides. Whole-mount brain imaging was performed using confocal laser-scanning microscopes (Leica SP5 or SP8, Leica microsystems, Wetzlar, Germany) using a 10X dry objective with 0.4 NA (10x HC PL APO CS, Leica microsystems No.11506285), a mechanical z-step of 2µm, and an x-y resolution of 512 x 512 pixels. For most species, imaging the whole brain required capturing 2 × 2 or 3 × 2 tiled stacks in the *x-y* dimensions (with 20% overlap) that were automatically merged in Leica Applications Suite Advanced Fluorescence software. In addition, where necessary brains were scanned from the posterior and anterior side to span the full *z*-dimension of the brain, due to low image quality with increasing *z*-distance. These two image stacks were then merged in Amira 3D analysis software 5.5 (FEI Visualization Sciences Group; custom module 'Advanced Merge'). Images of visual and olfactory projections in the MB (after 1.4) were obtained using the same microscopes and objective lens. The MB calyxes were scanned from the ventral surface of the brain at a resolution of 1024 x 1024 pixels (*x*,*y*) every 2µm (z). The "SYNORF1-cy2" neuropil staining was excited with an argon laser at 488nm wavelength, whereas traced projections with dextran-conjugated dyes (Tetramethylrhodamine and Alexa fluor 647) were excited at 561nm and 633nm respectively, with solid state lasers. To avoid crosstalk, each wavelength was sequentially scanned between lines, and emitted light was received on different detectors, a hybrid detector (Leica HyD, for cy2) and two standard PMTs. The *z*-dimension was scaled 1.52× to correct the artefactual shortening associated with the 10× air objective .

Images of brain slices (after 1.5-1.7) were obtained using the same confocal microscopes. To acquire images of the MB calyx and all surrounding Kenyon cell clusters, every brain section stained with "HRP-cy3" (after 1.6) was scanned at a resolution of 1024 x 1024 pixels (*x*,*y*) every 3µm (z) with a 10X dry objective (0.4 NA) and a solid state laser excitation at 561nm wavelength. Subsamples of the Kenyon cells cluster were then scanned at higher magnification with a 63X glycerol immersion objective with a 1.3 NA (63X HCX PL APO CS, Leica microsystems No. 11506194), and UV excitation at 405 nm wavelength. Images stacks measuring about 150 x 150 x 30 µm were produced for five randomly selected areas within the Kenyon cell clusters for each brain, at a resolution of 1024 x 1024 pixels (*x*,*y*) and a 1µm *z*-ztep. Images of presynaptic boutons stained with anti-SYNORF1 antibodies, and microglomeruli stained with Alexa-conjugated phalloidin (after 1.7) were acquired in a similar manner, using five randomly selected areas of each calyx with the same 63X glycerol objective. Boxes of approximately 125 x 125 x 25 µm were scanned to measure the density of

presynaptic boutons, and 95 x 95 x 25 μm for the microglomeruli density estimates, both at a resolution of 1024x1024 pixels every 1 μm along the depth (z).

For these analyses, the average densities from these subsampled regions were multiplied by the total volume or the Kenyon cell cluster/calyx to produce total numbers. Since the brain slices mounted in glycerol under cover slips were scanned with a 10X dry objective, there is an axial shift of the emitted light through different optical media resulting in mismatching refractive indices. To rescale images of brain sections, a correction factor was therefore assessed by scanning calibration beads (FocalCheck Microspheres, 15 μm, fluorescent green/orange/dark-red ring stains, F7235, Thermo Fisher Scientific) and calculating the ratio between measured z (depth) size and the actual size (15 μm). The average ratio obtained over 5 measurements of the microspheres (1.85) was used as correction factor of the z-dimension.

1.9 Supplementary Method 9: Confocal image analysis

For 3D segmentations from whole brain samples, confocal image stacks were saved as .lif files using the Leica application suite (RRID:SCR_013673) and imported in Advanced 3D Visualization and Volume Modelling software (Amira 5.4.3; Thermo Fisher Scientific, RRID:SCR_007353) to build virtual models of the neuropils. The z-dimension was rescaled by 1.52 when the mounting medium was methyl salicylate, whereas a correction of 1.85 was applied for brain slices scanned in glycerol (as described in 1.8). Image regions were assigned to anatomical structures with the *labelfield* module of the software, by defining separate outlines based on the brightness and contrast obtained in the different light channels (synaspsin: 488nm, neural tracing: 561 and 633 nm, HRP: 561nm). For large neuropil, approximately every fourth image was manually segmented and interpolated in the z-dimension across all images that contain the neuropil of interest, for smaller neuropils images were labelled at higher frequency. The volume of each 3D neuropil model was then extracted using the material statistics module of the Amira software. In total, the brains of 318 wild-caught individuals, representing 41 Heliconiini species, were reconstructed. For each of these brains, we reconstructed the volume of the mushroom body calyx, peduncle and lobes, two optic lobe neuropils, the medulla and ventral lobula, and the antennal lobe from one hemisphere, multiplying by two to get the total volume across the brain. In addition, we measured the total volume of the central brain (CBR), then subtracted the total volume of the mushroom body and antennal lobes to get an independent measure of brain size as an allometric control (rest-of-CBR, rCBR). For neural tracing experiment, the mushroom bodies and its sensory areas were segmented in 63 individuals belonging to 8 species (8 individuals/species with the exception of *H. doris* [7 individuals]) reared in controlled conditions, and for the Kenyon cell/synapse counting experiment the calyx and cell cluster of 50 individuals belonging to 6 species (approximately 8 individuals/species with the exception of *A. vanillae* [10 individuals for Kenyon cells]) were reconstructed from sectioned brains.

To assess the number of Kenyon cells across Heliconiini butterflies, the density of cell bodies was measured by generating cubes of 25 x 25 x 15 μm from each high magnification image stack of Kenyon cells clusters (5 per individual; described in 1.8) using ImageJ software (RRID:SCR_003070) and the Bio-Formats library (RRID:SCR_000450). The number of nuclei within each cube was

manually counted, omitting any cells with cell membranes not stained with HRP (see 1.6), and the average density was then multiplied by the volume of the whole Kenyon cells cluster around the MB (measured as described above) to obtain an estimated number of Kenyon cells per hemisphere for each individual.

The synaptic density within the calyx was measured from staining of presynaptic boutons with the anti-SYNORF1 antibody, using the ImageJ plugin 3D Object Counter (RRID:SCR_017066). The number of synapses was automatically counted within five boxes of 50 x 50 x 15 µm, adjusting intensity threshold to each image stack. Background noise was filtered by excluding all detected signals smaller than 10 voxels from the count, as manual measurements of synapse size suggested objects below this are unlikely to be genuine synapses. The density of microglomeruli in *Heliconius charithonia* and *Dryas iulia* was measured in a similar way as described for the Kenyon cells (above) in five of 25 x 25 x 10 µm cubes for each individual, generated from confocal image stacks of Alexa-conjugated phalloidin staining.

1.10 Supplementary Method 10: Electron microscopy sample preparation and imaging

To confirm cell count methods using immunohistochemistry and imaging of the Kenyon cell cluster, we obtained additional estimates using Electron Microscopy to image a cross section of the peduncle, to visualise Kenyon cell axons. For this purpose, butterflies were stunned/anaesthetised with Carbon dioxide prior to immersion in 2.5% glutaraldehyde in 0.1M cacodylate buffer at room temperature. Samples were then stored in a 4C fridge. Brains were subsequently removed, fixed and dehydrated using standard techniques. Briefly, treatments were made with osmium tetroxide (2% in cacodylate buffer), enbloc stained with uranyl acetate, dehydrated with ethanol, and infiltrated with propylene oxide, and then Epon resin mixture. Embedded blocks were polymerised at 60°C prior to sectioning on a Reichert Ultracut E. Transverse sections were taken of the peduncle to reveal axonal structure and imaged using a Tecnai T12 electron microscope (ThermoFisher UK) at 120 kV.

1.11 Supplementary Method 11: Ecological data

We scored three ecological traits for each Heliconiini species: the presence of pollen feeding, the number of host plant species exploited, and the degree of social roosting. We categorised pollen feeding as a binary trait, which was true for all *Heliconius,* except for *H. aoede,* and false for all other Heliconiini (3). Host plant use number was taken from the dataset collated by Kozak (4). Roosting behaviour was classed as either solitary, loose group, small group, or large group, taken from Brown (5).

1.12 Supplementary Method 12: Phylogenetic trees

All phylogenetic comparative analyses were conducted using a new phylogenetic tree of the Heliconiini generated from newly assembled genomes (6). To assemble additional genomes of *Heliconius erato cyrbia*, *H. melpomene rosina*, *H. m. amaryllis*, and *H. cydno galanthus*, short-read illumina data was downloaded from NCBI (National Centre for Biotechnology Information) and a reference-guided assembly approach adapted and extended from Lischer and Shimizu (7) adopted.

The strategy involves first mapping reads against a reference genome of a related species (*H. e. demophoon* for *H. e. cyrbia*; *H. m. melpomene* 2.5 for *H. m. rosina*, *H. m. amaryllis*; *H. cydno* for *H. cydno galanthus*) to reduce the complexity of *de novo* assembly within continuous covered regions, then later integrating reads with no similarity to the related genome.

Extra scaffolding procedures were implemented in order to improve the previous reference guided *de novo* assembly pipeline (7). Leveraging the very small genetic distances of these subspecies with their reference genomes, RNA-seq data from the reference species were downloaded from NCBI concatenated, corrected a normalized using *BBMap* v 38.79 (8) [target=20 maxdepth=20 mindepth=5]. These reads were mapped using *HISAT2* v 2.1.0 (9), and *P_RNA_scoffolder* (10). Following this step, *RaGOO* (11) was used [-T sr] for homology-based scaffolding and misassembly correction. *RaGOO* identifies structural variants and sequencing gaps, and accurately orders and orients *de novo* genome assemblies. *abyss-Sealer* (12) was then used with multiple *kmer*s [-k99 -k97 -k95 -k93 -k91 -k89 -k85 -k81 -k77 -k73 -k69 -k65 -k61 -k57] to finalise the assembly to close remaining gaps. After the genome assembly was completed, contaminants were identified using *BlobTools* v 1.1.1 (13) and removed from the final assemblies.

Single-copy orthologous genes in each genome were then identified using *BUSCO* (Benchmarking Universal Single-Copy Orthologs; v3.1.0 (14)). The Insecta set was selected in *OrthoDB* v 9 (odb9 1958 genes) using default parameters [-m genome]. For genes recovered as fragmented and missing a further mapping was implemented with *Exonerate*, using protein sequences found in other *Heliconius* species to deal with possible false discovery hits. For each species, all *BUSCO* genes found in a single copy were used for phylogenetic analysis.

Each nucleotide sequence *BUSCO* locus was aligned separately with *MACSE* v 2 (15), and all alignments were concatenated. From the concatenated alignment, gaps were removed using *Gblock* v 0.91b (16) [-t=c -b1=(#Nseq/2+1) -b2=(#Nseq/2+1) -b3=1 -b4=6 -b5=h] following Cicconardi et al. (2020; 2017). A maximum likelihood (ML) phylogenetic tree was performed using *IQ-TREE* v 2 (19), partitioning the supermatrix for each locus and codon position. *IQ-TREE* was run with the following settings: --runs 5 -m MFP. 5,000 ultrafast bootstrap replicates were conducted, resampling partitions and then sites within resampled partitions (20,21) to reduce false positives (-b 5000 --sampling GENESITE).

The Bayesian algorithm of *MCMCTree* (22) was implemented with approximate likelihood computation to estimate divergence times. First, branch lengths were estimated by ML, and then the gradient and Hessian matrix around these ML estimates was calculated in *MCMCTree* using the DNA supermatrix. Calibration nodes were constrained according to Cicconardi et al. (in prep). using a uniform distribution. To ensure convergence, the analysis was run for 10 x 100k generations after a 10M generations as burn-in, logging every 200 generations. *Tracer* v 1.7.1 (23) was used to check for convergence and ESS values were > 200.

2. Statistical analysis

2.1 Volumetric comparisons across Heliconiini

i) GLMs and allometric scaling

All volumetric measurements were log_{10}-transformed for statistical analyses. Where possible, analyses were conducted using individual-level data, though certain analyses required the use of species averages, calculated as the arithmetic mean. All analyses of wild data were run using the Helicoiniini phylogeny generated as described in 1.12, and were performed in R v 4.1.2. We ran a series of phylogenetic generalised linear mixed models (GLMM) with gaussian distributions using the R package *MCMCglmm* v 2.32 (24) to determine whether the scaling relationship between the mushroom body and the rest of the central brain (rCBR) changes between *Heliconius* and non-*Heliconius* Heliconiini, and between Heliconiini genera and subclades within *Heliconius* (25), including the effects of body size measurements and sex, where appropriate. We also included antennal lobe (AL) and medulla (ME) volumes to test whether increases in sensory neuropils, and therefore possibly increased sensory inputs, could provide a simple explanation of variation in mushroom body size. The best fitting model was then identified using Deviance Information Criterion (26). To determine whether any increases in MB size are driven by the expansion in a particular region of the mushroom body, we tested for variation in the scaling relationship between the mushroom body calyx and the lobe and peduncle between *Heliconius* and non-*Heliconius* species. We also assessed whether the mushroom bodies are unique in exhibiting expansion in *Heliconius* by testing whether the genus shows an increase in medulla, ventral lobula (vLO) or antennal lobe size, controlling for rCBR. Additionally, we tested for variation in rCBR size, using body size measurements as an allometric control. All models were checked for convergence using the *gelman.diag* function and for auto-correlation using the *autocorr* function provided in *MCMCglmm*, in addition to visually inspecting the trace plots. All models were run for 500,000 iterations, with a burn-in of 10,000 and a thinning factor of 500.

To support these analyses, we performed an additional set of analyses to examine allometric shifts between mushroom body size and rCBR. First, the function *sma* from the R package *smatr* v 3.4-8 was used to test for pairwise interspecific differences in the scaling relationship between mushroom body volume and rCBR across all species, testing for both differences in the slope and elevation of scaling relationships (27). For this analysis, only species with at least 8 individuals were included, reducing the dataset to 26 species, and the “robust” option was set to true for these analyses to minimise the influence of potential outliers (28).

ii) phylogenetic modelling using BAYOU

The R package *bayou* v 2.0 was used to identify regions of the Heliconiini tree showing evidence of a shift in the scaling relationship between mushroom volume and rCBR without *a priori* constraints (29). This method fits multi-optima Ornstein-Uhlenbeck models to phylogenetic comparative data, estimating the placement and magnitude of adaptive shifts. We compared three models: one with no shifts in either slope or elevation (all species are assumed exhibit the same allometric scaling

relationship), one allowing for shifts in elevation (a common allometric slope is assumed, but "grade-shifts" are allowed), and one allowing for shifts in both elevation and slope. Models were each run for 1,000,000 iterations, with a burn-in of 300,000 generations and their fits were compared using the *steppingstone* function in *bayou*, run for 10,000 generations with a burn-in of 3000 (30). The posterior probability cut-off for identifying a shift in the relationship between mushroom body and rCBR size was set at 0.5 following published recommendations (29,31,32).

iii) analysis of evolutionary rates and ancestral state reconstruction

To identify key periods of evolutionary change in a robust fashion, we used two different methods to test for shifts in the evolutionary rate of change in mushroom size within the Heliconiini, checking for consistency in the interpretations implied from the output of each method. First, we used *BayesTraits* v 3 to compare two independent contrast MCMC models of evolution, one allowing for a rate scaling parameter to vary across branches, and a second one where it is averaged across all branches (33,34). In the variable rates model, branch lengths are scaled to accommodate periods of increased trait change, and this scaling parameter provides an indication of evolutionary rate. Models were run for 110,000,000 iterations, with a burn-in of 10,000,000, and sampled every 10,000 iterations. Model fit was compared by calculating Log Bayes Factors using marginal likelihoods, calculated for each model using the stepping stone sampler (35) and sampling 100 stones for 10,000 iterations each, Log Bayes Factors are calculated as two times the difference in marginal log likelihoods of the two models, where a Log Bayes Factor <2 is interpreted as weak evidence for the more complex model, >2 as positive evidence, 5-10 as strong evidence, and >10 as very strong evidence. The output from the variable rates model was processed using the online tool (www.evolution.reading.ac.uk/BayesTraitsV4.0.0/BayesTraitsV4.0.0.html), to determine scaling factors for each node.

Second, we used a recently-published method that uses Brownian motion to model variations in evolutionary rate (36). Under this model, different branches of the tree are assumed to have different evolutionary rates, which also evolve via a Brownian process. Unlike the Bayesian approach implemented in *BayesTraits*, this method involves continuous (rather than discrete) changes in evolutionary rate between, or along, branches. This method has been implemented in the *multirateBM* function in the R package *phytools* v 0.7-90 (37). We used this method to estimate variations in the evolutionary rate of both mushroom body size. We then calculated the residual evolutionary rate of mushroom body size for each branch through a linear regression, using the *lm* function in R, of the estimated evolutionary rates of the mushroom body and rCBR size.

For these analyses of evolutionary rates, we used an expanded dataset that also included eight outgroup Lepidoptera species to better estimate the ancestral state at the *Heliconiini* root node. Neuropil volumes for the outgroup species were collected from published data (38–43), with the exception of *Bicyclus anynana* which was newly collected by S. Montgomery. Divergence times for outgroup species were taken from a recently published Lepidoptera phylogeny (44).

Finally, we also used two methods to estimate ancestral states for mushroom size and rCBR size at key internal nodes within the Heliconiini tree. First using *BayesTraits* v 3 we compared a

random walk and directional MCMC model of evolution in mushroom body size, controlling for rCBR, using the series of scaled trees generated from the post-burnin iterations of the variable rates model described above. The negative Log Bayes Factor (-16.84) for these two models indicated that a directional model of evolution was not supported. we then used the non-directional model and the scaled trees to estimate values for MB size and rCBR size at key internal nodes. Second, we used the *fastAnc* function in the *phytools* package to estimate the maximum likelihood ancestral states for mushroom body size and rCBR size at each node in the Heliconiini tree. The ancestral state density plots in Figure 2B we generated using the distributions of ancestral states estimated using *BayesTraits* v 3.

iii) Re-analysis of Snell-Rood et al. (2020) dataset

To corroborate our findings, we re-analysed a published, independently collected, dataset of neuroanatomy for 41 species of North American butterfly, which included *H. charithonia* and the Heliconiini *Agraulis vanillae*. Using this dataset, we ran the BAYOU analysis (2.1.ii), and the evolutionary rates analyses (2.1.iii) as described above. We used a recently published phylogeny of North American butterflies to perform these analyses (45).

2.2 Variation in Kenyon cell number, calyx synapses and microglomeruli

All count data were log transformed to meet the assumptions of being normal distributed. We tested for interspecific variation in Kenyon cell number, and separately Kenyon cell density, with generalised linear models (GLMs) with species as a fixed effect. We tested for variation in total Kenyon cell number, and separately Kenyon cell density, between *Heliconius* and non-*Heliconius* individuals using generalised linear mixed models (GLMMs) with a gaussian distribution, with membership in the *Heliconius* genus as a fixed effect and species as a random effect. We then explored whether the relationship between Kenyon cell number and calyx volume varies between taxa using a GLM with Kenyon cell number and species as fixed effects, and whether it varies between *Heliconius* and non-*Heliconius* using a GLMM with *Heliconius* membership as fixed effects and species as a random effect. A similar approach was adopted for synaptic data: interspecific differences between synapse density and total synapse number were examined separately using GLMs with species as a fixed effect. Differences between *Heliconius* and non-*Heliconius* individuals were also tested using GLMMs with a Gaussian distribution and *Heliconius* membership as a fixed effect and species as a random effect. Finally, variation in microglomeruli density between *Heliconius charithonia* and *Dryas iulia* was tested using a GLM with species as a fixed effect.

All models were built in R using the *glm* function for GLMs and the *glmer* function from package *lme4* v 1.1-30 for GLMMs. Diagnostics were assessed using the package *DHARMa* v 0.4.4 for R (46). All pairwise differences were assessed by calculating the estimated marginal means using the function *emmeans* in the R package *emmeans* v1.7.0, correcting for multiple comparisons using the Tukey test (47). We also independently assessed whether the relationship between Kenyon cell number and calyx volume varies between species using the function *sma* from the R package *smatr* v 3.4-8. We tested for pairwise differences between species in both the slope and elevation of the

scaling relationship between these traits. The “robust” option was set to true for these analyses to minimise the influence of potential outliers (28).

2.3 Variation in sensory input into the calyx

Visual and olfactory calyx volumes were log transformed for better normal distribution. Overall differences between *Heliconius* and non-*Heliconius* individuals in visual and olfactory calyx volume were analysed separately using GLMMs with *Heliconius* membership as a fixed factor and species as a random factor. We then determined whether the variation was higher in the visual or olfactory calyx by comparing the effect sizes of *Heliconius* membership using the *eff_size* function in the R package *emmeans* v 1.7.0. To test whether the scaling relationship between the visual and olfactory regions of the calyx differs between species, we used a GLM with olfactory calyx volume and species as fixed effects. The building of models and testing for pairwise differences follows the methods set out under section 2.2. We also assessed whether the scaling relationship between the visual and olfactory calyces differs between species using the function *sma* from the R package *smatr* v 3.4-8, testing for pairwise differences in both slope and elevation. The “robust” option was set to true for these analyses to minimise the influence of potential outliers (28).

Finally, the R package *bayou* v 2.0, was used to identify evidence of phylogenetic shifts in the scaling relationship between the visual and olfactory regions of the calyx within the eight sampled species (see 2.1.ii) (29). We compared three models: one with no shifts in either slope or elevation (all species are assumed exhibit the same allometric scaling relationship), one allowing for shifts in elevation (a common allometric slope is assumed, but “grade-shifts” are allowed), and one allowing for shifts in both elevation and slope. Models were each run for 1,000,000 iterations, with a burn-in of 300,000 generations and their fits were compared using the *steppingstone* function in *bayou*, run for 10,000 generations with a burn-in of 3000 (30). The posterior probability cut-off for identifying a shift in the relationship between mushroom body and rCBR size was set at 0.5 following published recommendations (29,31,32).

2.4 Testing ecological hypotheses

To determine whether key shifts in mushroom body size co-occurred with transitions in pollen feeding state we estimated ancestral states (presence/absence of pollen feeding) in internal nodes. The state of the discrete trait of pollen feeding at internal nodes was estimated using three different methods: (1) MCMC stochastic character mapping in *phytools* for 1000 simulations, (2) maximum likelihood using the *ace* function in the R package *ape* v 5.5 (48), and (3) maximum parsimony using the *asr_max_parsimony* function in the R package *castor* v 1.7.0 (49). The *fitDiscrete* function from the R package *geiger* 2.0.7 (50) was used to determine the best fitting transition model between equal rates, symmetric and all rates differ. ‘Equal Rates’ was the best fitting model and was used for all downstream analyses.

Finally, we explored whether certain ecological factors could explain variation in mushroom body size across Heliconiini using phylogenetic generalised linear mixed models (GLMM) in *MCMCglmm* v 2.32 (24) . We took the best fitting model explaining variation in mushroom body size

(see 2.1) and then included ecological factors – the degree of social roosting, host plant number/generalism, and pollen feeding (which was tested by removing the *Heliconius* factor with which it is almost perfectly confounded), to test whether these improve the fit of the model. All models were checked for convergence using the *gelman.diag* function and for auto-correlation using the *autocorr* function provided in *MCMCglmm*, in addition to visually inspecting the trace plots. All models were run for 500,000 iterations, with a burn-in of 10,000 and a thinning factor of 500.

3. Behavioural experiments

3.1 Animals

The behavioural experiments, using colour cues, were carried out on captive-reared butterflies between in either Gamboa, Panama, or, during travel restrictions related to the Covid-19 pandemic, in greenhouses at the University of Bristol, UK. All experiments were run using *Heliconius erato* and *Dryas iulia*, with additional *Heliconius melpomene* for the positive patterning experiment. All experiments were conducted using freshly-eclosed, naiive individuals.

In Gamboa, all larvae were reared from stocks established with locally caught, wild butterflies using the insectaries at the Smithsonian Tropical Research Institute in Gamboa, Panama. Stock butterflies were kept in 2 x 2 x 3 m mesh cages in ambient conditions with natural light. Larvae were reared in mesh pop-ups and were provided with fresh leaves daily. *H. erato* and *D. iulia* were reared on *P. biflora*, and *H. melpomene* on *P. triloba*. Training and testing of butterflies were conducted in 2 x 2 x 3 m mesh cages in ambient conditions under natural light. A single *Psychotria elata*, with all flowers removed, was placed in the rear right corner of these cages as a roosting site.

In Bristol, *D. iulia* and *H. erato* pupae were shipped to Bristol from the Stratford Butterfly Farm (UK) and Costa Rica Entomological Supply (Costa Rica). Pupae were kept in a climate-controlled greenhouse at 30°C and 80% humidity until emergence. The biconditional discrimination assay was conducted using adults that emerged from these pupae.

3.2 Positive patterning

The positive patterning experiment was conducted at the Smithsonian Tropical Research Institute insectaries in Gamboa, Panama, 2019. For this assay, stock populations of *Dryas iulia*, *H. erato* and *H. melpomene* were established from wild-caught individuals around Gamboa, Panama. Stock populations were kept in outdoor cages in ambient conditions. Adult stock butterflies were fed daily with a sugar-protein solution (20% sugar, 5% Vertark Critical Care Formula, 75% water, w/v) mixture and had access to *Psiguria* and *Lantana* flowers. Larvae of both species were fed with *Passiflora biflora* leaves. The positive patterning assay was conducted using only captive-reared butterflies from these stock populations. The day after eclosion, experimental individuals were introduced to a pre-training cage containing only white feeders filled with sugar-protein solution. Individuals were kept in this pre-training environment for two full days to acclimatise to using the feeders before beginning training.

After pre-training, butterflies were subject to an initial preference test between three types of artificial feeders: yellow, purple, yellow + purple. Butterflies were introduced to a testing cage containing 12 artificial feeders (4 yellow, 4 purple and 4 yellow + purple) arranged randomly with at least 6.5 cm between feeders on each side. To ensure that butterflies responded to visual cues only, feeders in the testing cages were empty. Butterflies were deprived of food from 12:00 the day prior to testing to encourage feeding during the trials. The preference test lasted for four hours and was filmed using mounted GoPro Hero 5 cameras. The film was then reviewed to count the number of feeding

attempts per individual on each colour. A feeding attempt was only counted if the butterfly landed on the feeder and probed it with its proboscis.

After the initial preference test, butterflies were placed in training cages containing 12 feeders (4 yellow, 4 purple and 4 yellow + purple). The yellow + purple feeders contained a food reward while the yellow and purple feeders were filled with a saturated solution of quinine, serving as an aversive stimulus. These feeders were arranged randomly each morning. Butterflies kept in training cages for eight days and could freely sample the feeders for the entire period. Following the training period, butterfly feeding preferences were re-tested, following the same protocol as the initial preference test.

3.3. Biconditional discrimination

The biconditional discrimination trials were conducted in greenhouses at the University of Bristol in 2020. For this assay. The biconditional discrimination assay was conducted using adults that emerged from these commercially sourced pupae. The day after eclosion experimental individuals were introduced to a pre-training cage containing only white feeders filled with sugar-protein solution. Individuals were kept in this pre-training environment for two full days to acclimatise to using the feeders before beginning training.

The biconditional discrimination assay used artificial feeders of four different colour combinations: red + blue, purple + yellow, red + yellow, purple + blue (Figure 5). Following the pre-training period, butterflies were placed in a cage containing four empty feeders of each colour combination to test for their initial preference, following the testing protocol described for the positive patterning assay. Butterflies were then randomly assigned to one of two training regimes.

For the first training regime, butterflies were introduced to a cage containing four feeders of each colour combination, with the purple + yellow and blue + red feeders containing a food reward and the yellow + red and blue + purple combination filled with the aversive quinine solution (Figure 5). Each colour was therefore evenly represented between rewarded and punished feeders. Accordingly, butterflies could not solve the task by learning a single colour but needed to learn specific combinations of colours. The training regime for the second group was the reverse of the first, with yellow + red and purple + blue feeders rewarded and purple + yellow and blue + red punished. The eight-day training period and subsequent testing followed the same protocol as the positive patterning assay.

3.4 Long term memory

Long-term memory (LTM) and reversal learning (RL) experiments, using colour cues, were carried out on captive-reared butterflies between January and April 2019 in Gamboa, Panama. Individuals were transferred to a pre-training cage one day after eclosion. Here, butterflies were fed solely with white artificial feeders containing a sugar-protein solution (20% sugar, 5% Vertark Critical Care Formula, 75% water, w/v) for two days (from 08:00 to 12:00) to familiarise them with the use of artificial feeders.

After pre-training, butterflies were introduced to a testing cage to determine initial feeding preferences between purple and yellow. Testing cages contained 12 purple and 12 yellow feeders randomly in a 4 X 6 grid, with 6.5 cm between feeders on each side. To ensure that butterflies

responded exclusively to visual cues, feeders in the testing cages were empty. Preference testing lasted for four hours from 08:00 to 12:00 and was filmed from above using a GoPro Hero 5 camera mounted on a tripod. Butterflies were individually numbered on their wings for identification using a permanent marker. The film was then reviewed to count the number of feeding attempts per individual on each colour, with up to 30 attempts recorded per individual. A feeding attempt was only counted if the butterfly landed on the feeder and probed it with its proboscis.

Butterflies were then trained to associate a food reward with their non-favoured colour, based on the results of their initial preference test. For butterflies that initially preferred purple, the training cage contained yellow feeders containing a sugar-protein solution, and purple feeders containing a saturated quinine solution, an aversive stimulus. The opposite arrangement was employed for individuals that initially preferred yellow. This training period lasted for four full days. After training, butterfly preferences were re-tested, following the same protocol as the initial preference test, to verify that individuals had indeed acquired the colour-food association.

After the trained preference test, individuals participating in the long-term memory assay were placed for eight days in a cage identical to the pre-training cage, containing only white feeders filled with a sugar-protein solution. The deprivation of colour stimuli for eight days allowed for testing the long-term memory retention of the colour-food association acquired during the training period, and ensured that long-term memory was being tested rather than short-term or mid-term memory. A period of eight days was chosen because *Heliconius* are known to maintain their foraging routes over periods ranging from weeks to months, during which time a pollen resource could be unproductive for several days due to competition or damage, but ultimately rewarding over the long term. Butterflies were then subject to a third preference test to determine if the learned preference was maintained, following the same protocol as the initial preference test. When subsequently analysing the data, individuals that exhibited less than 50% accuracy during the initial trained preference test, and therefore did not appear to have learned the food-colour association, were removed from the dataset. In total, 1 out of 48 *H. erato* individuals, and 4 of 63 *Dryas iulia,* were removed from the dataset.

3.5 Statistical analysis

Learning performance in the positive patterning and biconditional discrimination assays was analysed with generalised linear mixed models (GLMMs) using the *glmer* function from the R package *lme4* v 1.1-27.1 (51). These models included species and training as fixed effects (in addition to their interaction), with an individual-level random effect. For the biconditional discrimination assay, since different butterflies were trained to different colour combinations, training regime was also included as a fixed effect. However, training regime was non-significant and therefore removed from the model. To test for interspecific differences in the drop in performance between the initial preference test and the long-term memory test, an interaction between species and trial was also included. Diagnostics for these models were assessed using the package *DHARMa* (46). All post hoc comparisons were made by obtaining the estimated marginal means using the R package *emmeans* v 1.7.0 and were corrected for selected multiple comparisons using the Tukey test (47).

4. Supplementary Notes

4.1 Supplementary Note 1: Clade and pairwise differences in mushroom body scaling

Within the Heliconiini, mushroom body expansion did not occur solely along the branch leading to *Heliconius*, but rather occurred in a stepwise series along the phylogeny (Figure S1). Among the non-*Eueides* and non-*Heliconius* Heliconiini, *Dryadula phaetusa* shows an expansion of the mushroom bodies (Figure S1A). The entire *Eueides* genus shows mushroom body expansion comparable to that in *Dryadula phaetusa*, with a further expansion in the '*lybia'* subclade (Figure S1B). This culminates in a further, even more marked, expansion in *Heliconius* (Figure S1C). Notably, the loss of pollen feeding in *Heliconius aeode* is not associated with a corresponding decrease in mushroom body volume (Figure S1C; yellow). GLMM analysis that include phylogenetic grouping (outgroup genus or subclade within *Heliconius*) as a factor shows mushroom body size, controlling for rCBR, antennal lobe and medulla size, as internally consistent both within *Heliconius* and amongst the outgroup Heliconiini (Table S1). However, MB volume in *Dryadula phaetusa* is not significantly different to either the *Heliconius* clades or the other outgroups, while *Eueides* only differ from *melpomene*-group *Heliconius (*Table S1). Uncorrected pairwise comparisons, however, do show *Dryadula phaetusa* and *Eueides* as having significantly larger MBs than several outgroup Heliconiini, and smaller MBs than some Heliconius.

Supporting these results, pairwise allometric analysis with the R package *smatr* indicates that while the slope of the allometric relationship between rCBR and mushroom body volume does not differ between Heliconiini species, there are significant differences in elevation (Table S2). In general, relative mushroom body volume is consistent throughout *Heliconius* and larger than in the outgroups, congruent with the results of the phylogenetic GLMM above. *Eueides* and *Dryadula phaetusa* have MB volumes intermediate between *Heliconius* and the other outgroups, while *H. telesiphe*, *H. doris* and *H. aoede* have smaller mushroom bodies than several other *Heliconius* species.

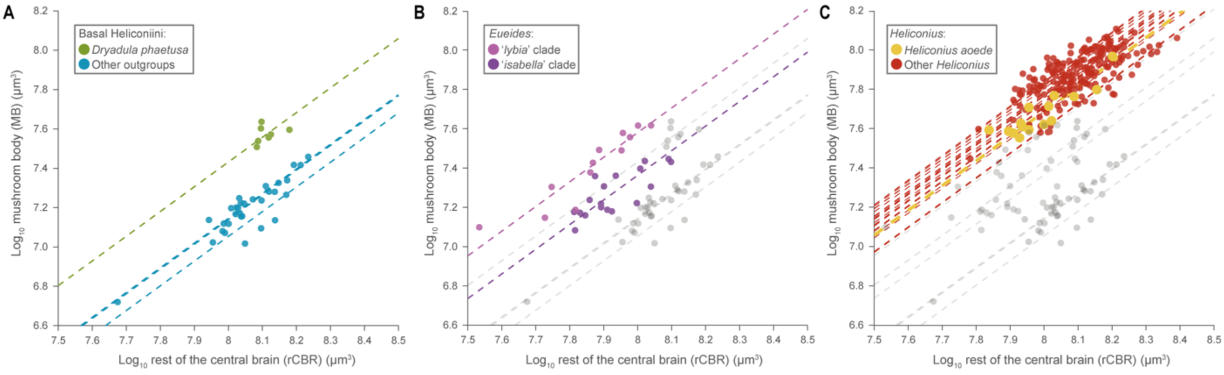

Figure S1: Stepwise increases in mushroom body (MB) across the Heliconiini phylogeny. (**A**) Amongst the non-*Heliconius* and non-*Euedies* Heliconiini, *Dryadula phaetusa* show enlarged MBs. (**B**) *Eueides* show an upshift in MB size relative to non-*Dyradula* basal Heliconiini and within the genus, the '*lybia'* clade shows a further enlargement. (**C**) *Heliconius* show a further, even larger, increase in mushroom body size. Notably, the non-pollen-feeding *Heliconius aoede* (yellow) does not show an decrease in mushroom body size relative to other *Heliconius*. Regression lines show (A,B) species and (B) main clades. Points indicate individuals. Source Data: Heliconiini_neuro_individuals.csv.

Table S1. Pairwise comparisons between Heliconiini clades for mushroom body size, derived from a phylogenetic generalised linear mixed model including the size of the antennal lobe, the medulla and the rest of the central brain as fixed effects. Z-ratio shown above the diagonal and p-values below, corrected for multiple comparisons using Tukey's test (* = p<0.05; ** = p<0.01; *** = p<0.001).

		**Non-*Heliconius* Heliiconini**							***Heliconius* clade**						
		Philaethria	***Dryadula***	***Podotricha***	***Dryas***	***Dione***	***Agraulis***	***Eueides***	**erato**	**sara/sapho**	**aeode**	**doris**	**wallacei**	**silvaniforms**	**melpomene**
	**No. Species**	**1**	**1**	**1**	**1**	**1**	**1**	**5**	**7**	**7**	**1**	**1**	**2**	**5**	**7**
	**No. individuals**	**7**	**7**	**2**	**14**	**4**	**11**	**30**	**60**	**41**	**15**	**9**	**11**	**31**	**76**
**Non-*Heliconius* Heliconiini**	***Philaethria***		2.532	-0.888	0.787	-0.051	0.370	2.223	4.285**	-4.252**	3.694*	3.267	-4.093**	-4.586***	4.638***
	Dryadula	0.386		1.782	1.895	-2.369	-1.937	0.274	-1.801	-1.789	1.349	0.956	-1.740	-2.162	-2.261
	Podotricha	1.000	0.885		-0.111	-0.820	-0.419	1.293	0.0448*	-3.353	2.885	2.398	-3.240	-3.684*	3.764*
	Dryas	1.000	0.830	1.000		-0.750	-0.337	-1.438	-3.478*	-3.454*	2.971	2.534	-3.369*	-3.825**	-3.911***
	Dione	1.000	0.503	1.000	1.000		0.609	-2.308	-4.346**	-4.293**	3.782*	-3.385*	-4.150**	-4.688***	-4.710***
	Agraulis	1.000	0.8065	1.000	1.000	1.000		-1.911	-3.974**	-3.908**	-3.484*	-3.024	-3.804*	-4.316**	-4.373**
	Eueides	0.613	1.000	0.991	0.978	0.549	0.8208		3.013	-2.919	2.328	1.680	-2.722	-3.346	-3.395*
Heliconius	**erato**	0.0015**	0.876	0.0448*	0.0335*	0.0015**	0.0055**	0.134		-0.002	-0.656	-1.238	0.110	-0.682	-0.806
	**sara/sapho**	0.0017**	0.881	0.0501	0.0363*	0.0015**	0.0071**	0.170	1.000		-0.634	01.203	0.108	-0.664	0.778
	**aeode**	0.158*	0.987	0.184	0.149	0.0115*	0.0329*	0.534	1.000	1.000		0.628	-0.534	-1.345	-1.461
	**doris**	0.0652	1.000	0.482	0.384	0.0452*	0.130	0.923	0.994	0.996	1.000		-1.210	-2.016	-2.167
	**wallacei**	0.0034**	0.902	0.0707	0.0477*	0.0027**	0.0106*	0.266	1.000	1.000	1.000	0.995		0.878	1.021
	**silvaniforms**	0.0004***	0.658	0.0164*	0.0097**	0.0002***	0.0013**	0.0511	1.000	1.000	0.988	0.758	1.000		0.270
	**melpomene**	0.0003***	0.585	0.0123*	0.0070**	0.0002***	0.0010**	0.0439*	1.000	1.000	0.974	0.654	0.999	1.000	

Table S2. Interspecific differences in elevation in the scaling relationship between the size of the mushroom bodies and the rest of the central brain. P-values above the diagonal are uncorrected, and below are corrected for multiple comparisons. Comparisons were made using the SMATR elevation test, tests are two sided. (. = p < 0.01; * = p < 0.05; ** = p < 0.01; *** p < 0.001; *Avan* = *Agraulis vanillae*; *Diul* = *Dryas iulia; Dpha* = *Dryadula phaetusa*; *Eisa* = *Eueides Isabella*; *Evib* = *Eueides vibilia*; *Haoe* = *H. aoede*; *Hcyd* = *H. cyndo chioneus*; *Hcyg* = *H. cydno galanthus*; *Hdor* = *H. doris*; *Herc* = *H. erato cyrbia*; *Herd* = *H. erato demophoon*; *Hhel* = *H. hecale*; *Hhew* = *H. hewitsoni*; *Hhim* = *H. himera*; *Hism* = *H. ismenius*; *Hmea* = *H. melpomene amaryllis*; *Hmel* = *H. melpomene melpomene*; *Hmer*; *H. melpomene rosina*; *Hnum* = *H. numata*; *Hpac* = *H. pachinus*; *Hsap* = *H. sapho*; *Hsar* = *H. sara*; *Htel* = *H. telesiphe*; *Htim* = *H. timareta*; *Hwal* = *H. wallacei*; *Pdid* = *Philaethria dido*).

		Heliconius																				**Non-*Heliconius* Heliconiini**					
		Hpac	***Hcyg***	***Hcyd***	***Htim***	***Hmel***	***Hmer***	***Hmea***	***Hhel***	***Hism***	***Hnum***	***Hwal***	***Hdor***	***Haoe***	***Hsap***	***Hhew***	***Hsar***	***Herc***	***Herd***	***Hhim***	***Htel***	***Evib***	***Eisa***	***Avan***	***Diul***	***Dpha***	***Pdid***
Heliconius	***Hpac***					.			.			*	***	***	**	.		*		**	***	***	***	***	***	***	***
	Hcyg					**		*	*			***	***	***	*	***		*		.	***	***	***	***	***	***	***
	Hcyd					**			*			**	***	***	*	***		**		**	***	***	***	***	***	***	***
	Htim					*		.	.			**	***	***	*	**		*		*	***	***	***	***	***	***	***
	Hmel						*	**			*						*		*		***	***	***	***	***	***	***
	Hmer								*			***	***	***	**	**		**		*	***	***	***	***	***	***	***
	Hmea								*	.		***	***	***	***	***		***		***	***	***	***	***	***	***	***
	Hhel																					***	***	***	***	***	***
	Hism										*								*		***	***	***	***	***	***	***
	Hnum											***	***	***	**	**		**		*	***	***	***	***	***	***	***
	Hwal						*	***			.		**				*	.	***	.	**	***	***	***	***	***	***
	Hdor	***	***	***	***		***	***			***			***	***	**	***	***	***	***		***	***	***	***	***	***
	Haoe	*	***	**	**		***	***			***		.		*		*	**	***	**	.	***	***	***	***	***	***
	Hsap							.					**						**		**	***	***	***	***	***	***
	Hhew		**					*											**		***	***	***	***	***	***	***
	Hsar												**							***	***	***	***	***	***	***	***
	Herc							*					***						**		***	***	***	***	***	***	***
	Herd											*	***	***						*	***	***	***	***	***	***	***
	Hhim							.					***				***				***	***	***	***	***	***	***
	Htel	**	***	***	***	.	***	***		*	***					***			***			***	**	***	***	***	***
**Non-*Heliconius* Heliconiini**	***Evib***	***	***	***	***	***	***	***	***	***	***	***	***	***	***	***	***	***	***	***	.			***	***		***
	Eisa	***	***	***	***	*	***	***	**	**	***	***	***	***	***	***	***	***	***	***				***	***		***
	Avan	***	***	***	***	***	***	***	***	***	***	***	***	***	***	***	***	***	***	***	***	***	***			***	*
	Diul	***	***	***	***	***	***	***	***	***	***	***	***	***	***	***	***	***	***	***	***	***	***			***	
	Dpha	***	***	***	***	***	***	***	**	***	***	***	***	***	***	***	***	***	***	***				***	***		***
	Pdid	***	***	***	***	***	***	***	***	***	***	***	***	***	***	***	***	***	***	***	***	***	***			***	

4.2 Supplementary Note 2: Sex effects on mushroom body size

Heliconius females have significantly larger mushroom bodies than males (pMCMC < 0.001), whereas no such sex effect was identified in the outgroup Heliconiini (pMCMC = 0.690) (Figure S2). This difference could potentially reflects sex differences in pollen foraging behaviour, with females tending to collect more pollen than males (52,53).

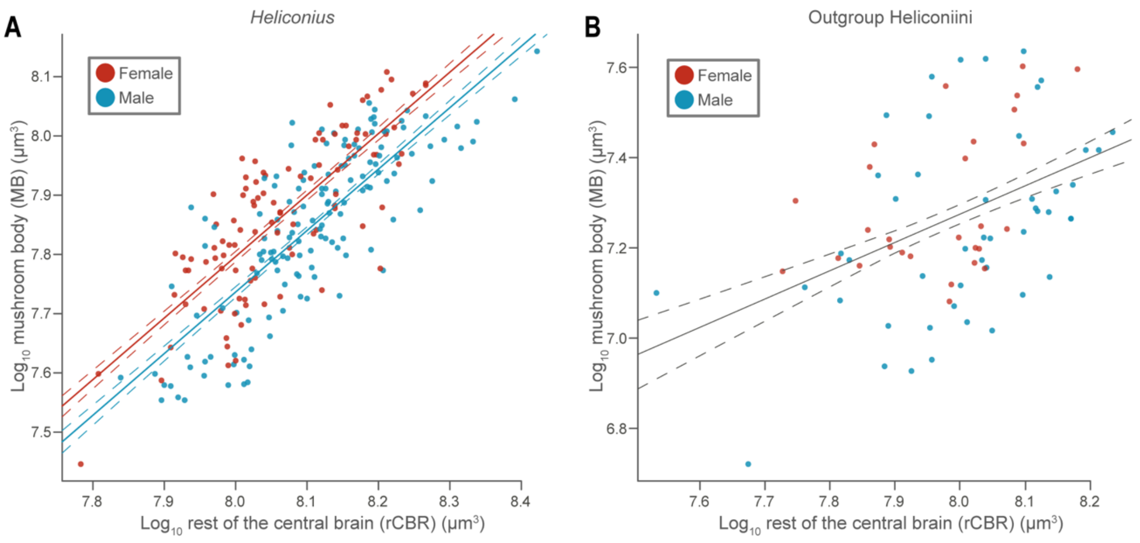

Figure S2: Sexual dimorphism in mushroom body size in *Heliconius*. (**A**) Female *Heliconius* have larger mushroom bodies than males (pMCMC < 0.001). (**B**) There is no sex effect for outgroup Heliconiini (pMCMC = 0.690). Data points represent individuals ($n_{Heliconius}$ = 243; $n_{Outgroup\ Heliconiini}$ = 75). The central line shows predicted means using a fitted linear model, with dashed lines showing standard errors. Source Data: Heliconiini_neuro_individuals.csv.

4.3 Supplementary Note 3: Additional results on mushroom body evolutionary rates

Our findings of a marked expansion the mushroom body in *Heliconius* are corroborated by our re-analysis of an independently collected neuroanatomical dataset of 41 species of North American butterflies, including *H. charithonia* and the Heliconiini *Agraulis vanillae* (54). Using the R package *bayou v* 2.0, we identified phylogenetic shifts in the scaling relationship between the mushroom body and the rest of the central brain. The best fitting model permitted shifts in elevation specifically (marginal likelihoods: elevation shifts = 25.874; both = 7.003; none = 7.656) and identified two shifts with a posterior probability greater than 0.5 (Figure 2A,B). We resolved a distinct upshift in mushroom body volume in *Heliconius* (post. prob. = 0.90; Figure S3; Table S4), despite an overall decrease in mushroom body volume in the Nymphalidae as a whole (excluding *Danaus plexippus*) (post. prob. = 0.69; Figure S3A,B; Table S4). Similarly, *H. charithonia* stands out amongst the sampled butterflies as having an exceptionally elevated rate of mushroom body evolution when analysed using both the *multirateBM* function in the R package *phytools* (Figure S3C,D) and the variable rates model in *BayesTraits* v 3 (Bayes Factor = 3.542; Figure S4C,D).

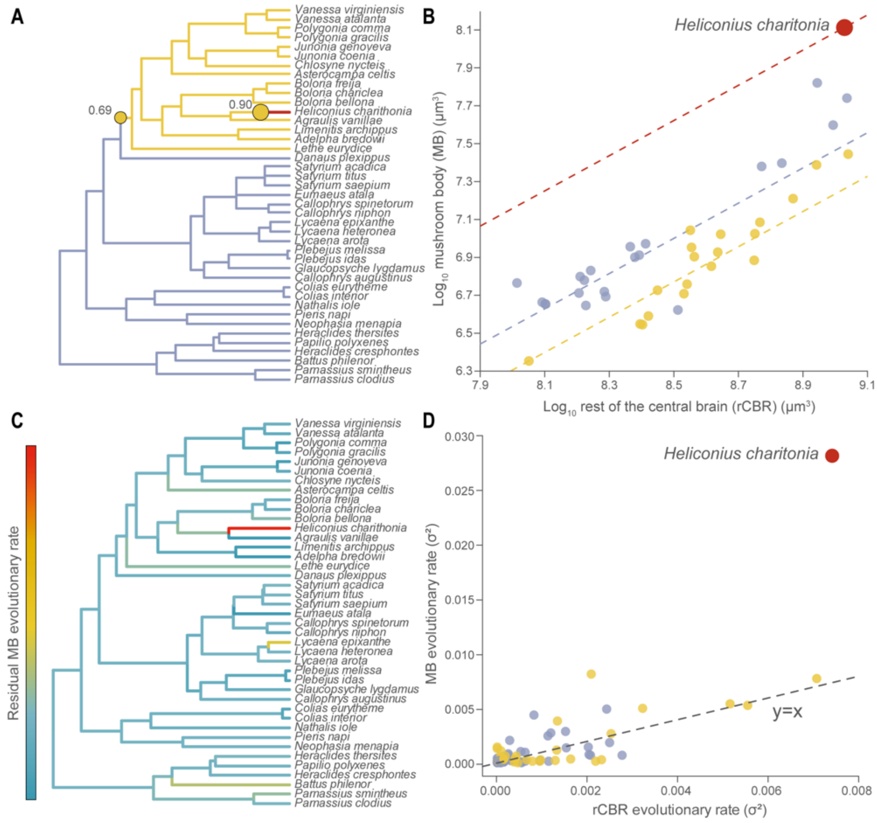

Figure S3: *Heliconius* stand out as having markedly expanded and increased evolutionary rate of the mushroom bodies even in a phylogenetically wide sampling of butterflies. (**A** and **B**) Phylogenetic shifts in the scaling relationship between the volume of the mushroom body (MB) and the rest of the central brain (rCBR) across 41 North American butterfly species (posterior probability > 0.5). Despite a decrease in MB volume in the Nymphalidae (excluding *Danaus plexippus*) (blue), *Heliconius* exhibit a dramatic increase in MB volume relative to this phylogenetically wide sampling of butterflies. (**C** and **D**) This increase in MB size is associated with a pronounced increase in the estimated evolutionary rate of MB volume, controlling for the evolutionary rate of rCBR volume. Source Data: SnellRood.csv and Earl2021.trees.

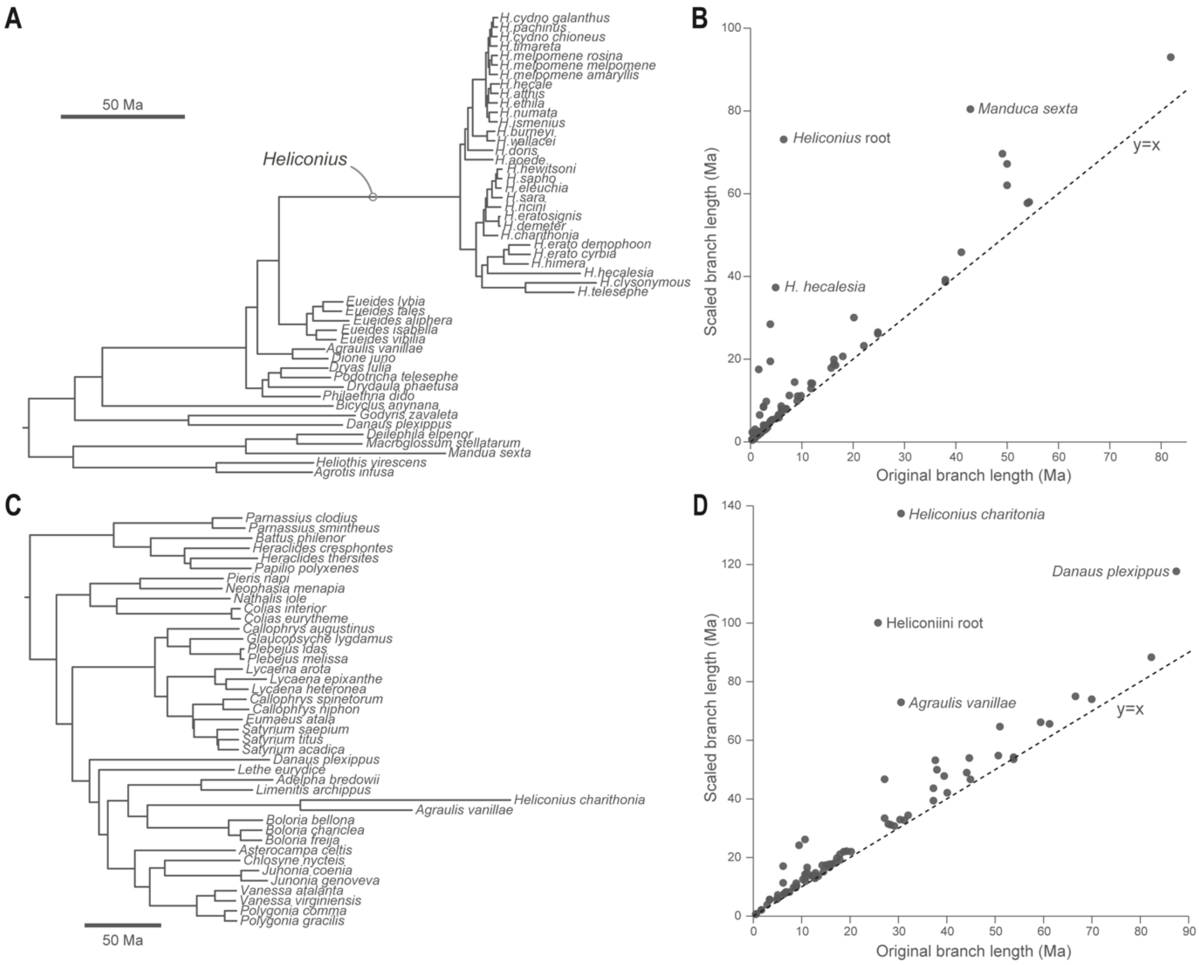

Figure S4: Increased evolutionary rate of the mushroom body in *Heliconius*. Estimates of the evolutionary rate of the mushroom body (MB) (controlling for the size of the rest of the central brain) produced from multiple iterations of a variable rates models in *BayesTraits* v 3, using originally ultrametric trees. (**A**) Consensus scaled tree for 41 Heliconiini species and eight outgroup Lepidoptera, with (**B**) scaled branch length plotted against original branch lengths. (**C**) Consensus scaled tree for 41 North American butterfly species, including *H. charithonia* and *Agraulis vanilla*, generated using neuroanatomical data from Snell-Rodd et al. (2020) and the consensus tree in Earl et al. (2021), with (**D**) scaled branch lengths plotted against original branch lengths. Increased branch lengths in (A) and (C) and deviance from y = x in (B) and (D) indicate an estimated increase in the evolutionary rate of the MB. Source Data: BayesMBrCBRindividuals.txt, BayesMBrCBRmeans.txt and Heliconiini+Outgroups.trees.

4.4 Supplementary Note 4: Additional neuroanatomical detail

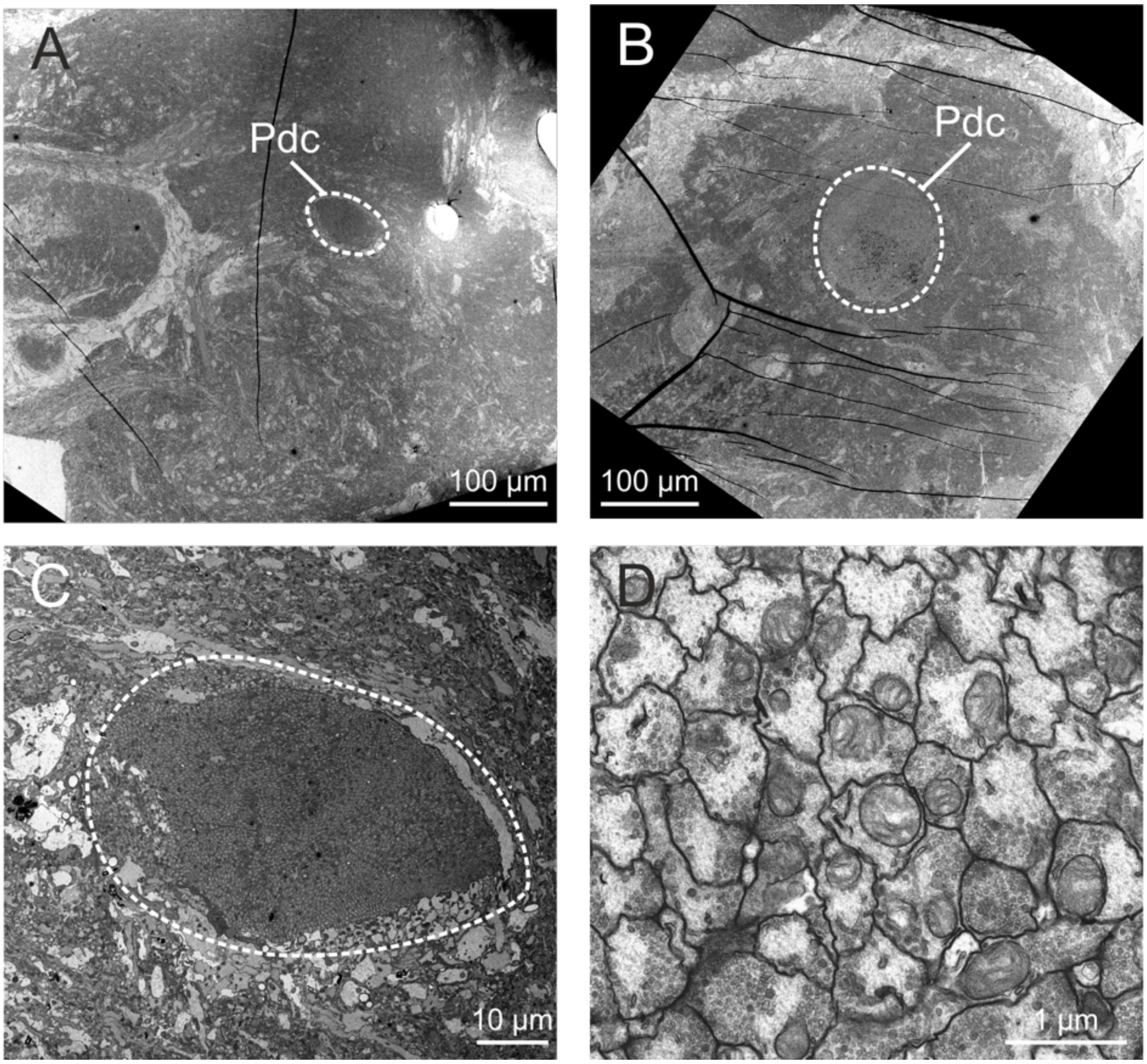

Figure S5: An example of the electron microscopy imaging. (A) low magnification of orthogonally sectioned brains showing the peduncle (Pdc) in *Dryas iulia*, and **(B)** *Heliconius erato*. **(C)** Mid-magnification cross section through at *Dryas iulia* and **(D)** high magnification image of cross-sectioned Kenyon cell axons running through the peduncle. Total Kenyon cell counts were estimated by measuring the area of the cross section at mid magnification and the density of Kenyon cell axons at high magnification, and multiplying the two estimates. Images are representative of all samples (2 indivdiuals per species).

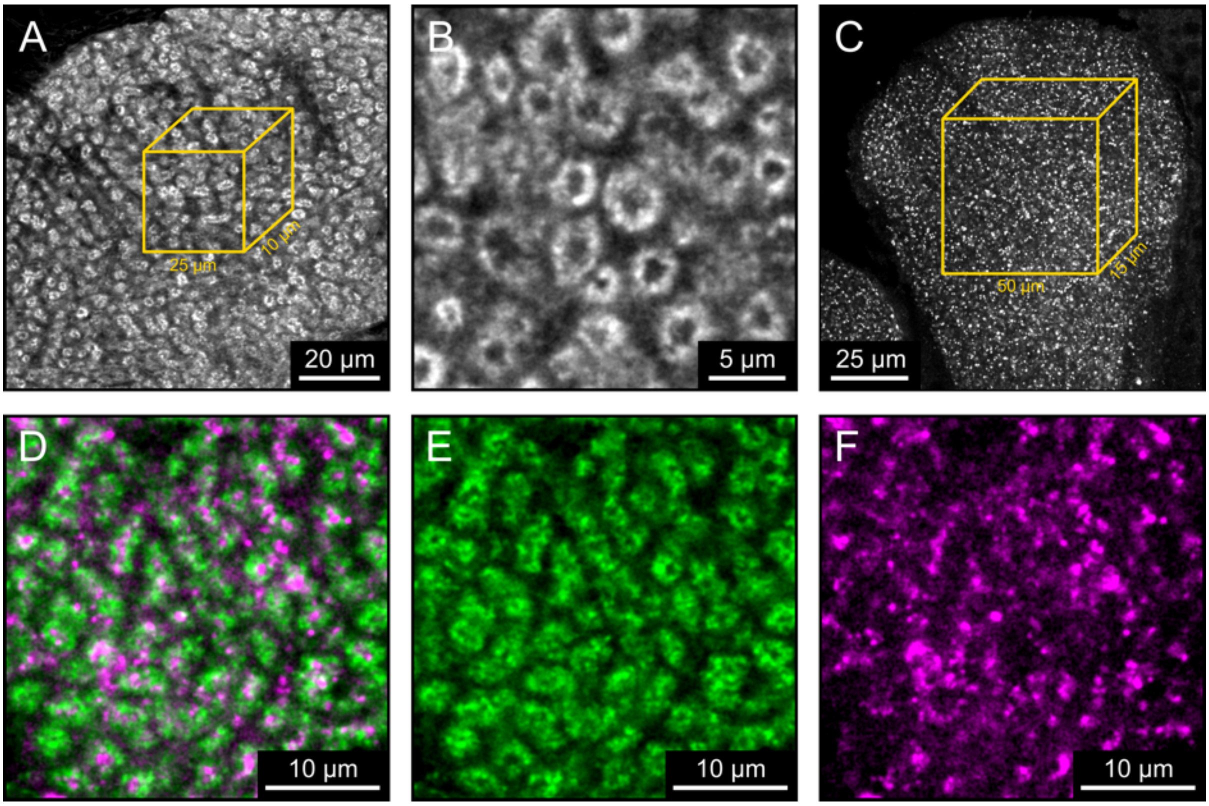

Figure S6: An example of the microglomeruli staining. (A) An optical section through a portion of the calyx of *H. charithonia* after staining of the Kenyon cells dendrites with phalloidin conjugated dye. A cube of 25x25x10 µm was extracted from the image stack to manually count the microglomeruli structure at **(B)** high magnification. **(C)** An optical section through a portion of the calyx of *H. hecale* after staining of the synaptic boutons with anti-Synorf1 antibody. Synaptic boutons were automatically counted within cubes of 50x50x15 µm. **(D)** Combined labelling of **(E)** Kenyon cell dendrites and **(F)** synaptic terminals in the calyx of *H. erato*. The microglomeruli structure appear to be innervated by more than a single postsynaptic bouton. Images are representative of data presented in Figure 3, see figure 3 for sample sizes/species.

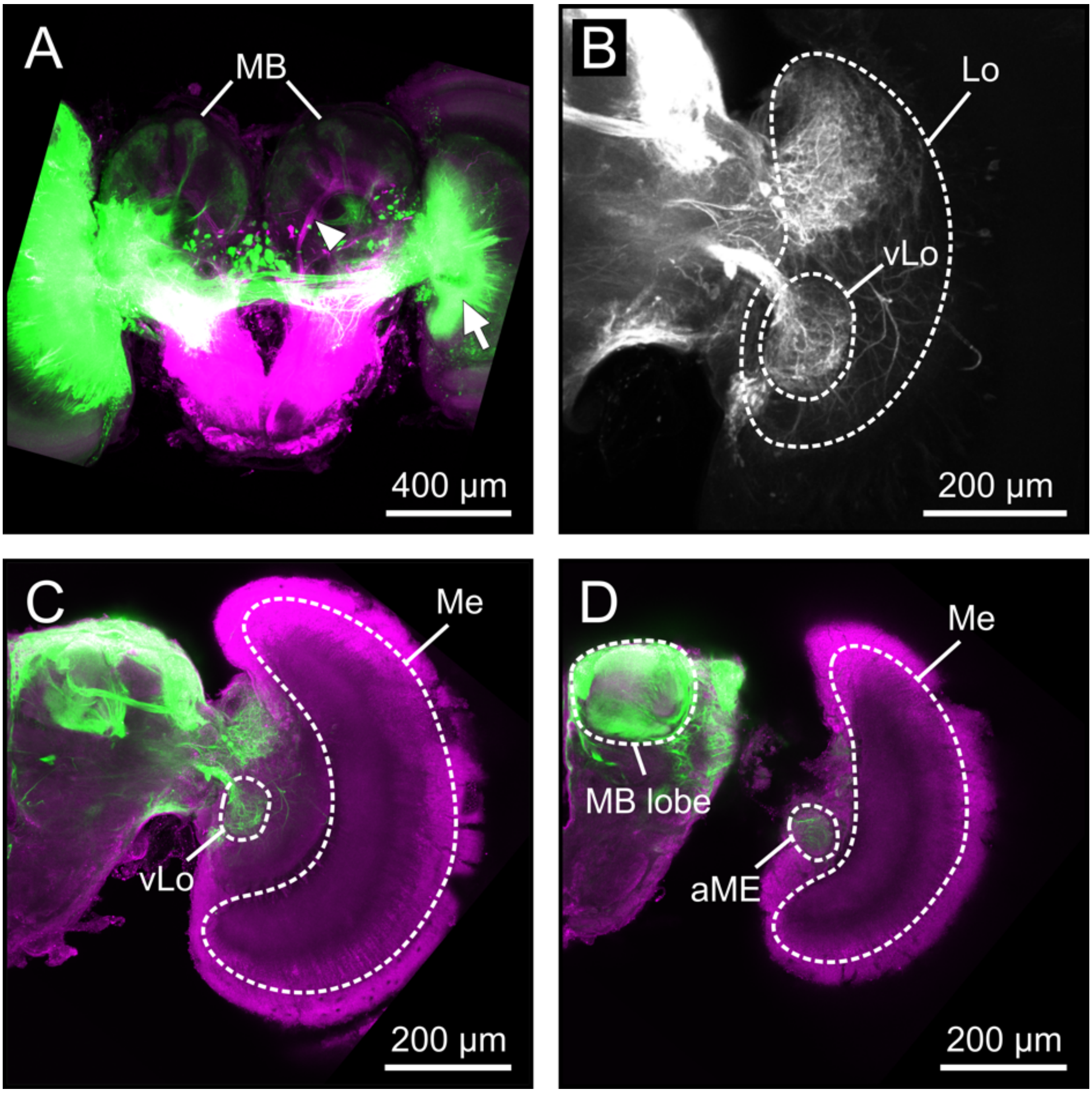

Figure S7: Identifying visual neuropils that innervate the mushroom body calyx. (A) Optical section through the brain of *H. melpomene* after injection of a fluorescent tracer in the optic lobes (green) and the antennal lobe (magenta). Both sensory neuropil have projection into the mushroom bodies. The arrowhead shows a major track of olfactory projection neurons, whereas the complete arrow shows the injection site in the optic lobes. **(B)** An optical section through the optic lobe after injection of a fluorescent tracer in the mushroom body calyx itself. The visual calyx is mainly arborized by projection neurons from the ventral lobe of the lobula (vLo). **(C)** A large tract of visual projection neurons leave the vLo as ab optical relay and run through the central brain to the MB. **(D)** At the dorsal surface, the accessory medulla (aMe) also send projection to the MB calyx. Images are shown from a representative individual.

Table S3. Interspecific differences in total Kenyon cell number. Pairwise comparisons from generalised linear model testing the effect of species on Kenyon cell number, corrected for multiple comparisons using Tukey's test. T-ratio values shown above the diagonal and p-values below. (* = p <0.05; ** = p < 0.01; *** = p < 0.001).

	Dryas iulia	*Agraulis vanillae*	*E. isabella*	*H. hecale*	*H. melpomene*	*H. hortense*
Dryas iulia		-2.159	-7.833***	-21.324***	-22.868***	-23.201***
Agraulis vanillae	0.2774		-10.416***	-24.636***	-26.263***	-26.614***
E. isabella	<0.0001***	<0.0001***		-13.491***	-15.035***	-15.368***
H. hecale	<0.0001***	<0.0001***	<0.0001***		-1.544	-1.877
H. melpomene	<0.0001***	<0.0001***	<0.0001***	0.6385		0.333
H. hortense	<0.0001***	<0.0001***	<0.0001***	0.4294	0.9994	

Table S4. Interspecific differences in the scaling relationship between Kenyon cell number and calyx volume. Pairwise comparisons from generalised linear model testing the effect of species on mushroom body calyx volume, controlling for Kenyon cell number, corrected for multiple comparisons using Tukey's test. T-ratio values shown above the diagonal and p-values below. (* = p <0.05; ** = p < 0.01; *** = p < 0.001).

	Dryas iulia	*Agraulis vanillae*	*E. isabella*	*H. hecale*	*H. melpomene*	*H. hortense*
Dryas iulia		3.315*	4.115*	-0.144	1.598	0.112
Agraulis vanillae	0.0215*		1.732	-1.039	0.627	-0.739
E. isabella	0.0022*	0.5187		-3.023*	-0.255	-2.361
H. hecale	1.0000	0.9020	0.0453*		6.058***	-0.859
H. melpomene	0.6044	0.9884	0.9998	<0.0001***		5.320***
H. hortense	1.0000	0.9758	0.1929	0.9541	<0.0001***	

Table S5. Interspecific differences in the scaling relationship between Kenyon cell number and calyx volume using SMATR. Pairwise comparisons for SMATR analysis testing for differences in elevation in the relationship between KC number and calyx volume, corrected for multiple comparisons. Test stat values shown above the diagonal and p-values below. (* = p <0.05; ** = p < 0.01; *** = p < 0.001).

	Dryas iulia	*Agraulis vanillae*	*E. isabella*	*H. hecale*	*H. melpomene*	*H. hortense*
Dryas iulia		2.423	16.512***	1.0212	12.405**	3.815
Agraulis vanillae	0.852		3.948	0.239	6.437	0.858
E. isabella	0.0007***	0.514		4.298	3.065	0.01133
H. hecale	0.996	1.000	0.442		30.671***	0.788
H. melpomene	0.006**	0.155	0.714	<0.0001***		14.0160**
H. hortense	0.543	0.999	1.000	0.999	0.0027**	

4.5 Supplementary Note 5: Evolution of ventral lobula size

Using the R package *bayou v* 2.0, we tested for phylogenetic shifts in the scaling relationship between the ventral lobula and the rest of the central brain. Although the best fitting model permitted shifts in elevation specifically (marginal likelihoods = 19.434) this was only marginally higher than the null model (marginal likelihood = 18.797) resulting in only weak evidence supporting these grade shifts (Bayes Factor = 1.274). Nevertheless, the model allowing for shifts in intercept did not identify any shifts associated with *Heliconius*, but rather size reductions of the ventral lobula at the base of *Eueides* and the *Philaethria dido* branch (Figure 2A,B). These size reductions appear to be associated with a slight increase in the evolutionary rate of the ventral lobula in *Eueides* (Figure S8C). *H. eratosignis* and *H. demeter* do show elevated rates of ventral lobula evolution (Figure S8C), but this is likely due to a minor, but significant, divergence in ventral lobula volume over very short branch lengths, as they otherwise cluster within *Heliconius* in an allometric plot (Figure 8B).

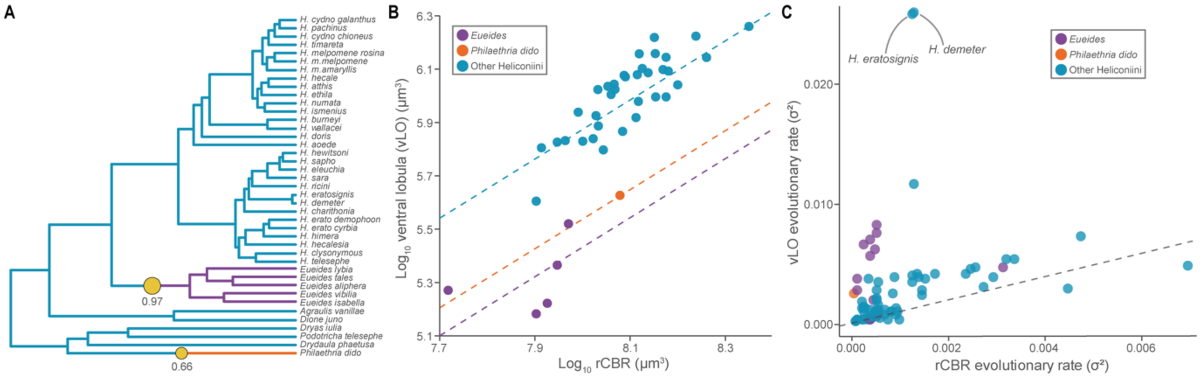

Figure S8: Variation in the volume and evolutionary rate of the ventral lobula across the Heliconiini. (**A-B**) Phylogenetic shifts in the scaling relationship between the volume of the ventral lobula (vLO) and the rest of the central brain (rCBR) across 41 Heliconiini species (posterior probability > 0.5). vLO size reductions are identified in *Eueides* and *Philaethria dido*. (**C**) branch specific rates in vLO size plotted against rCBR size. Source Data: Heliconiini.trees and Heliconiini_neuro_species.csv.

4.6 Supplementary Note 6: Variation in sensory innervation to the mushroom body calyx

Table S6. Pairwise comparisons from generalised linear model testing the effect of Species on visual calyx volume, controlling for olfactory calyx volume, corrected for multiple comparisons using Tukey's test. Data were analysed with generalised linear models treating species and training as fixed effects along with an individual-level random effect. Posthoc comparisons were made by deriving the estimated marginal means and using a two-sided t-test, correcting for multiple comparisons using Tukey's test. T-ratio values shown above the diagonal and p-values below. Comparisons were made using the SMATR elevation test, tests are two sided. * indicates p<0.05, ** indicates p<0.01, *** indicates p<0.001.

	Dryadula phaetusa	*Dryas iulia*	*Agraulis vanillae*	*E. isabella*	*H. doris*	*H. hecale*	*H. melpomene*	*H. hortense*
Dryadula phaetusa		5.658***	7.490***	2.994	-12.981***	-16.843***	-19.083***	-19.345***
Dryas iulia	<0.0001***		3.352*	-3.501*	-16.835***	-16.423***	-18.028***	-17.767***
Agraulis vanillae	<0.0001***	0.0297*		-5.951***	-17.537***	-16.505***	-17.930***	-17.630***
E. isabella	0.0741	0.0197*	<0.0001***		-15.787***	-17.550***	-19.563***	-19.448***
H. doris	<0.0001***	<0.0001***	<0.0001***	<0.0001***		-4.501***	-6.599***	7.596***
H. hecale	<0.0001***	<0.0001***	<0.0001***	<0.0001***	0.0009***		-2.474	4.246**
H. melpomene	<0.0001***	<0.0001***	<0.0001***	<0.0001***	<0.0001***	0.2285		1.801
H. hortense	<0.0001***	<0.0001***	<0.0001***	<0.0001***	<0.0001***	0.0021**	0.6222	

Table S7. Pairwise comparisons for SMATR analysis testing for differences in elevation in the relationship between visual calyx volume and olfactory calyx volume, corrected for multiple comparisons. * indicates p<0.05, ** indicates p<0.01, *** indicates p<0.001.

	Dryadula phaetusa	*Dryas iulia*	*Agraulis vanillae*	*E. isabella*	*H. doris*	*H. hecale*	*H. melpomene*	*H. hortense*
Dryadula phaetusa		6.0734	13.2093**	3.6579	180.6086***	359.9703***	115.4951***	231.3589***
Dryas iulia	0.3208		2.5785	1.0077	37.0090***	143.9715***	18.6955***	85.5057***
Agraulis vanillae	0.00777**	0.960		4.8189	58.2198***	154.6392***	31.1753***	92.8329***
E. isabella	0.7997	1.000	0.5505		119.6457***	217.1556***	45.1626***	140.0605***
H. doris	0.0001***	0.0001***	0.0001***	0.0001***		17.7068***	1.2914	17.3266***
H. hecale	0.0001***	0.0001***	0.0001***	0.0001***	0.0001***		5.5056	25.5943***
H. melpomene	0.0001***	0.0004***	0.0001***	0.0001***	0.9997	0.415		0.4976
H. hortense	0.0001***	0.0001***	0.0001***	0.0001***	0.00088***	0.0001***	1.000	

4.7 Supplementary Note 7: Reconstructing pollen feeding evolution

We used three different methods to estimate the discrete trait of pollen feeding at internal nodes in the Heliconiini tree. Both MCMC stochastic character mapping (Figure S9A) and maximum likelihood (Figure S9B) estimates indicate pollen feeding almost certainly arose only once in the Heliconiini, at the base of *Heliconius*, and was secondarily lost in the 'Neruda' clade, including *H. aoede*. Maximum parsimony, however, suggests it is equally likely that pollen feeding arose once and was lost once or arose twice within *Heliconius* (Figure S9C).

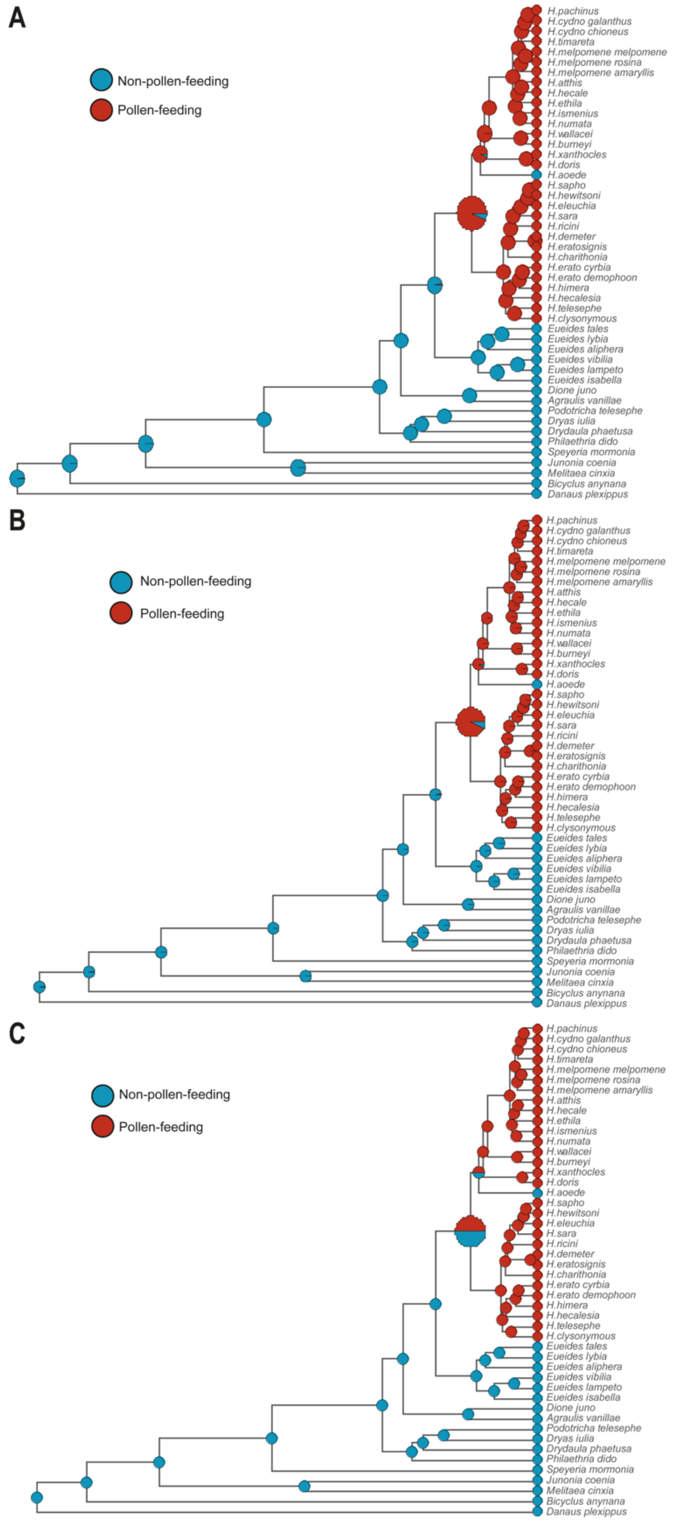

Figure S9. Pollen feeding likely arose once in the Heliconiini, at the base of *Heliconius*. Ancestral state of pollen feeding in the internal nodes of the Heliconiini tree estimated using (**A**) MCMC stochastic character mapping, (**B**) maximum likelihood, and (**C**) maximum parsimony. The colour of the circles represents the probability of pollen feeding at a given node. *Heliconius* root node is enlarged for clarity. Source Data: Heliconiini+Outgroups.trees and pollenfeeding.csv

4.8 Supplementary Note 8: Visual positive patterning learning in *Heliconius melpomene*

In addition to *Dryas iulia* and *H. erato*, we also assessed the visual positive patterning learning ability in *H. melpomene*, following identical protocols. *H. melpomene* was capable of solving this task (Table S8), with an accuracy comparable to *H. erato* and similarly superior to *Dryas iulia* (Table S8; Figure S10).

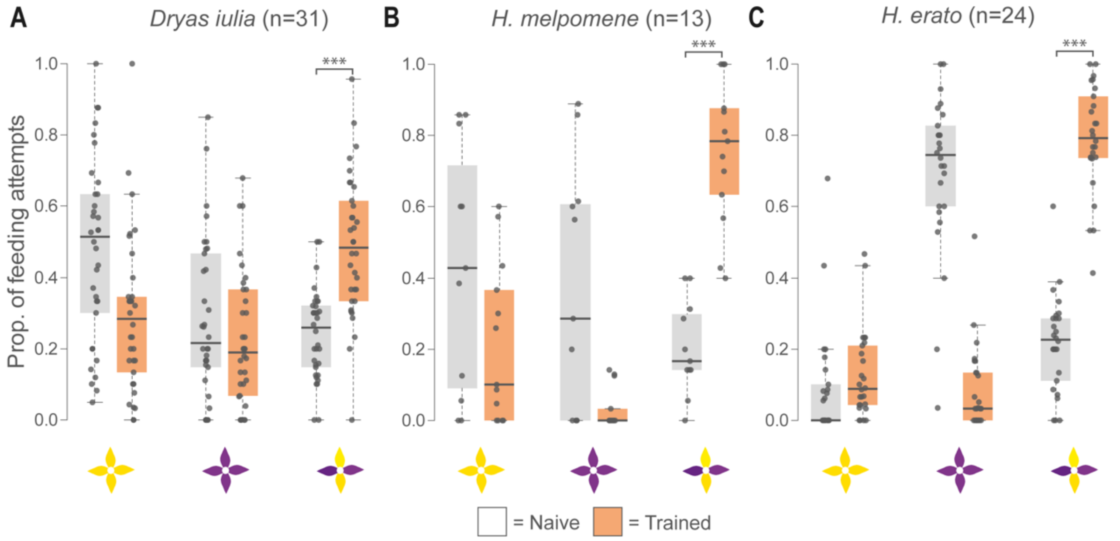

Figure S10: *Heliconius erato* and *Heliconius melpomene* outperform *Dryas iulia* in a visual positive patterning task after eight days' training. (**A**) *Dryas iulia* was able to solve this visual positive patterning learning task, but its accuracy was lower than both (**B**) *H. melpomene* and (**C**) *H. erato*. The box encompasses two middle quartiles, with central line showing median. Whiskers extend to the furthest data point within 1.5 times the interquartile range. * = p < 0.05; ** = p < 0.01; *** = p < 0.001.

Table S8. Visual positive patterning learning in Heliconiini butterflies. Selected pairwise comparisons, corrected multiple comparisons using the Tukey test, between the proportion of correct feeding attempts by *Dryas iulia* (n = 31), *H. erato* (n = 24) and *H. melpomene* (n = 13) before and after training. Source Data: pospatdata.csv

Contrast	Estimate	SE	Z ratio	P value
Dryas iulia naive – *Dryas iulia* trained	-1.018	0.111	-9.195	<0.0001***
H. erato naive – *H. erato* trained	-2.567	0.157	-16.405	<0.0001***
H. melpomene naive – *H. melpomene* trained	-2.593	0.270	-9.610	<0.0001***
Dryas iulia naive – *H. erato* naive	0.230	0.117	1.300	0.785
Dryas iulia naive – *H. melpomene* naive	0.611	0.268	2.277	0.203
H. erato naive – *H. melpomene* naive	0.381	0.285	1.336	0.765
Dryas iulia trained – *H. erato* trained	-1.320	0.167	-7.902	<0.0001***
Dryas iulia trained – *H. melpomene* trained	-0.964	0.222	-4.333	0.0002***
H. erato trained – *H. melpomene* trained	0.356	0.236	1.509	0.659

5. Supplementary References

1. Ott SR. Confocal microscopy in large insect brains: Zinc–formaldehyde fixation improves synapsin immunostaining and preservation of morphology in whole-mounts. J Neurosci Methods. 2008; 172(2):220–30.

2. Fahrbach SE, Van Nest BN. Synapsin-based approaches to brain plasticity in adult social insects. Curr Opin Insect Sci. 2016; 18:27–34.

3. Gilbert, LE. Biodiversity of a Central American *Heliconius* community: pattern, process, and problems. In: *Plant-animal interactions: evolutionary ecology in tropical and temperate regions*. 1991. p. 403–27.

4. Kozak, KM. Macroevolution and phylogenomics in the adaptive radiation of Heliconiini butterflies [PhD Thesis]. University of Cambridge, UK; 2015.

5. Brown KS. The biology of *Heliconius* and related genera. Annu Rev Entomol. 1981; 26(1):427–57.

6. Cicconardi F, Milanetti E, de Castro ÉCP, Mazo-Vargas A, Van Belleghem SM, Ruggieri AA, et al. Evolutionary dynamics of genome size and content during the adaptive radiation of Heliconiini butterflies. BioRxiv 2022. Available from: http://biorxiv.org/lookup/doi/10.1101/2022.08.12.503723

7. Lischer HEL, Shimizu KK. Reference-guided de novo assembly approach improves genome reconstruction for related species. BMC Bioinformatics. 2017; 18(1):474.

8. Bushnell B. BBMap: a fast, accurate, splice-aware aligner. Lawrence Berkeley National Lab.(LBNL), Berkeley, CA (United States); 2014.

9. Kim D, Paggi JM, Park C, Bennett C, Salzberg SL. Graph-based genome alignment and genotyping with HISAT2 and HISAT-genotype. Nat Biotechnol. 2019; 37(8):907–15.

10. Zhu BH, Xiao J, Xue W, Xu GC, Sun MY, Li JT. P_RNA_scaffolder: a fast and accurate genome scaffolder using paired-end RNA-sequencing reads. BMC Genomics. 2018; 19(1):175.

11. Alonge M, Soyk S, Ramakrishnan S, Wang X, Goodwin S, Sedlazeck FJ, et al. RaGOO: fast and accurate reference-guided scaffolding of draft genomes. Genome Biol. 2019; 20(1):224.

12. Paulino D, Warren RL, Vandervalk BP, Raymond A, Jackman SD, Birol I. Sealer: a scalable gap-closing application for finishing draft genomes. BMC Bioinformatics. 2015; 16(1):230.

13. Laetsch DR, Blaxter ML. BlobTools: Interrogation of genome assemblies. F1000Research. 2017; 6:1287.

14. Simão FA, Waterhouse RM, Ioannidis P, Kriventseva EV, Zdobnov EM. BUSCO: assessing genome assembly and annotation completeness with single-copy orthologs. Bioinformatics. 2015; 31(19):3210–2.

15. Ranwez V, Douzery EJP, Cambon C, Chantret N, Delsuc F. MACSE v2: Toolkit for the Alignment of Coding Sequences Accounting for Frameshifts and Stop Codons. Mol Biol Evol. 2018; 35(10):2582–4.

16. Talavera G, Castresana J. Improvement of phylogenies after removing divergent and ambiguously aligned blocks from protein sequence alignments. Syst Biol. 2007; 56(4):564–77.

17. Cicconardi F, Krapf P, D'Annessa I, Gamisch A, Wagner HC, Nguyen AD, et al. Genomic Signature of shifts in selection in a subalpine ant and its physiological adaptations. Mol Biol Evol. 2020; 37(8):2211–27.

18. Cicconardi F, Marcatili P, Arthofer W, Schlick-Steiner BC, Steiner FM. Positive diversifying selection is a pervasive adaptive force throughout the *Drosophila* radiation. Mol Phylogenet Evol. 2017; 112:230–43.

19. Minh BQ, Schmidt HA, Chernomor O, Schrempf D, Woodhams MD, von Haeseler A, et al. IQ-TREE 2: new models and efficient methods for phylogenetic inference in the genomic era. Mol Biol Evol. 2020; 37(5):1530–4.

20. Gadagkar SR, Rosenberg MS, Kumar S. Inferring species phylogenies from multiple genes: Concatenated sequence tree versus consensus gene tree. J Exp Zoolog B Mol Dev Evol. 2005; 304B(1):64–74.

21. Seo TK, Kishino H, Thorne JL. Incorporating gene-specific variation when inferring and evaluating optimal evolutionary tree topologies from multilocus sequence data. Proc Natl Acad Sci. 2005; 102(12):4436–41.

22. Yang Z. PAML 4: Phylogenetic Analysis by Maximum Likelihood. Mol Biol Evol. 2007; 24(8):1586–91.

23. Rambaut A, Drummond AJ. Tracer v. 1.7. Program and documentation. 2009.

24. Hadfield JD. MCMC methods for multi-response generalized linear mixed models: the MCMCglmm *R* package. J Stat Softw 2010. Available from: http://www.jstatsoft.org/v33/i02/

25. Baker J, Meade A, Pagel M, Venditti C. Nothing wrong with the analysis of clades in comparative evolutionary studies: a reply to Poe et al. Syst Biol. 2021; 70(1):197–201.

26. Berg A, Meyer R, Yu J. Deviance information criterion for comparing stochastic volatility models. J Bus Econ Stat. 2004; 22(1):107–20.

27. Warton DI, Duursma RA, Falster DS, Taskinen S. smatr 3– an R package for estimation and inference about allometric lines. Methods Ecol Evol. 2012; 3(2):257–9.

28. Taskinen S, Warton DI. Robust estimation and inference for bivariate line-fitting in allometry. Biom J. 2011; 53(4):652–72.

29. Uyeda JC, Harmon LJ. A novel bayesian method for inferring and interpreting the dynamics of adaptive landscapes from phylogenetic comparative data. Syst Biol. 2014; 63(6):902–18.

30. Fan Y, Wu R, Chen MH, Kuo L, Lewis PO. Choosing among partition models in bayesian phylogenetics. Mol Biol Evol. 2011; 28(1):523–32.

31. Uyeda JC, Pennell MW, Miller ET, Maia R, McClain CR. The evolution of energetic ccaling across the vertebrate tree of life. Am Nat. 2017; 190(2):185–99.

32. Khabbazian M, Kriebel R, Rohe K, Ané C. Fast and accurate detection of evolutionary shifts in Ornstein–Uhlenbeck models. Methods Ecol Evol. 2016; 7(7):811–24.

33. Pagel M. Inferring the historical patterns of biological evolution. Nature. 1999; 401(6756):877–84.

34. Venditti C, Meade A, Pagel M. Multiple routes to mammalian diversity. Nature. 2011; 479(7373):393–6.

35. Xie W, Lewis PO, Fan Y, Kuo L, Chen MH. Improving marginal likelihood estimation for bayesian phylogenetic model selection. Syst Biol. 2011; 60(2):150–60.

36. Revell LJ. A variable-rate quantitative trait evolution model using penalized-likelihood. PeerJ. 2021; 9:e11997.

37. Revell LJ. phytools: an R package for phylogenetic comparative biology (and other things): *phytools: R package*. Methods Ecol Evol. 2012; 3(2):217–23.

38. El Jundi B, Huetteroth W, Kurylas AE, Schachtner J. Anisometric brain dimorphism revisited: Implementation of a volumetric 3D standard brain in *Manduca sexta*. J Comp Neurol. 2009; 517(2):210–25.

39. Kvello P. Digital, three-dimensional average shaped atlas of the *Heliothis virescens* brain with integrated gustatory and olfactory neurons. Front Syst Neurosci. 2009; 3.

40. Heinze S, Reppert SM. Anatomical basis of sun compass navigation I: The general layout of the monarch butterfly brain. J Comp Neurol. 2012;520(8):1599–628.

41. Stöckl AL, Ribi WA, Warrant EJ. Adaptations for nocturnal and diurnal vision in the hawkmoth lamina: visual adaptations in the Hawkmoth lamina. J Comp Neurol. 2016;524(1):160–75.

42. Montgomery SH, Ott SR. Brain composition in *Godyris zavaleta*, a diurnal butterfly, reflects an increased reliance on olfactory information. J Comp Neurol. 2015; 523(6):869–91.

43. Adden A, Wibrand S, Pfeiffer K, Warrant E, Heinze S. The brain of a nocturnal migratory insect, the Australian Bogong moth. J Comp Neurol. 2020; 528(11):1942–63.

44. Kawahara AY, Plotkin D, Espeland M, Meusemann K, Toussaint EFA, Donath A, et al. Phylogenomics reveals the evolutionary timing and pattern of butterflies and moths. Proc Natl Acad Sci. 2019; 116(45):22657–63.

45. Earl C, Belitz MW, Laffan SW, Barve V, Barve N, Soltis DE, et al. Spatial phylogenetics of butterflies in relation to environmental drivers and angiosperm diversity across North America. iScience. 2021; 24(4):102239.

46. Hartig, F, Hartig, M.F. Package 'DHARMa'. Vienna Austria R Dev Core Team. 2017.

47. Lenth R, Singmann H, Love J, Buerkner P, Herve M. Emmeans: Estimated marginal means, aka least-squares means. R Package Version. 2018;1(1):3.

48. Paradis E, Schliep K. ape 5.0: an environment for modern phylogenetics and evolutionary analyses. Bioinformatics. 2019;35(3):526–8.

49. Louca S, Doebeli M. Efficient comparative phylogenetics on large trees. Bioinformatics. 2018; 34(6):1053–5.

50. Harmon LJ, Weir JT, Brock CD, Glor RE, Challenger W. GEIGER: investigating evolutionary radiations. Bioinformatics. 2008; 24(1):129–31.

51. Bates D, Sarkar D, Bates MD, Matrix L. The lme4 package. R Package Version. 2007; 2(1):74.

52. Cardoso MZ. Patterns of pollen collection and flower visitation by *Heliconius* butterflies in southeastern Mexico. J Trop Ecol. 2001; 17(5):763–8.

53. Boggs CL, Smiley JT, Gilbert LE. Patterns of pollen exploitation by Heliconius butterflies. Oecologia. 1981; 48(2):284–9.

54. Snell-Rood EC, Swanson EM, Espeset A, Jaumann S, Philips K, Walker C, et al. Nutritional constraints on brain evolution: Sodium and nitrogen limit brain size. Evolution. 2020; 74(10):2304–19.
