# Supplementary material for: Rapid expansion and visual specialisation of learning and memory centres in the brains of Heliconiini butterflies
Source: Nat Commun. 2023 Jul 7;14:4024. doi: 10.1038/s41467-023-39618-8 (PMC10328955; doi:10.1038/s41467-023-39618-8)
Supplement: Supplementary file 4 — Description of Additional Supplementary Files [file 41467_2023_39618_MOESM4_ESM.pdf]

Description of Additional Supplementary Files

Supplementary Data 1:
Description:

- Supplementary Data 1: Volumetric neuropil data for wild caught species: individual data
- Supplementary Data 2: Volumetric neuropil data for wild caught species: species means
- Supplementary Data 3: Kenyon cell number estimates
- Supplementary Data 4: Synapse number estimates
- Supplementary Data 5: Visual and olfactory calyx volumes
- Supplementary Data 6: Ecological data
- Supplementary Data 7: Behavioural data: positive patterning trials
- Supplementary Data 8: Behavioural data: biconditional discrimination trials
- Supplementary Data 9: Behavioural data: long term memory trials
