# Supplementary material for: Rapid expansion and visual specialisation of learning and memory centres in the brains of Heliconiini butterflies
Source: Nat Commun. 2023 Jul 7;14:4024. doi: 10.1038/s41467-023-39618-8 (PMC10328955; doi:10.1038/s41467-023-39618-8)
Supplement: Supplementary file 6 — Source Data [file 41467_2023_39618_MOESM6_ESM.zip › Couto, Young et al_Source Data 2/Couto, Young et al_Source Data list.docx]

Figure 1 uses the tree “Heliconiini.trees”.

Figure 2A uses the tree “Heliconiini.trees” and “Heliconiini_neuro_species.csv”.

Figure 2B uses “Heliconiini_neuro_individuals.csv” and “Heliconiini_neuro_species.csv”.

Figure 2C uses “Heliconiini.trees” and “Heliconiini_neuro_species.csv”.

Figures 2D-H uses “Heliconiini_neuro_species.csv”.

Figure 3D uses “KCdata.csv” and “Synapsedata.csv”.

Figure 3E uses “phalloidin_means.csv”.

Figure 3F uses “KCdata.csv”.

Figure 3G uses “Heliconiini_neuro_individuals.csv” and “Heliconiini_neuro_species.csv”.

Figures 4I-J use “Tracingdata.csv”.

Figure 5A uses “pospatdata.csv”.

Figure 5B uses “bicondiscdata.csv”.

Figure 5C uses “LTMdata.csv”.

Figures S1 and S2 use “Heliconiini_neuro_individuals.csv”.

Figure S3 uses “SnellRood.csv” and “Earl2021.trees”.

Figure S4 uses “BayesMBrCBRindividuals.txt”, “BayesMBrCBRmeans.txt” and “Heliconiini+Outgroups.trees”.

Figure S8 uses the tree “Heliconiini.trees” and “Heliconiini_neuro_species.csv”.

Figure S9 uses “Heliconiini+Outgroups.trees” and “pollenfeeding.csv”.

Figure S10 uses “pospatdata.csv”.
